# Benzylic C(sp^3^)–H fluorination

**DOI:** 10.3762/bjoc.20.137

**Published:** 2024-07-10

**Authors:** Alexander P Atkins, Alice C Dean, Alastair J J Lennox

**Affiliations:** 1 University of Bristol, School of Chemistry, Bristol, BS8 1TS, U.K.

**Keywords:** benzylic, C–H functionalization, fluorination, photoredox catalysis

## Abstract

The selective fluorination of C(sp^3^)–H bonds is an attractive target, particularly for pharmaceutical and agrochemical applications. Consequently, over recent years much attention has been focused on C(sp^3^)–H fluorination, and several methods that are selective for benzylic C–H bonds have been reported. These protocols operate via several distinct mechanistic pathways and involve a variety of fluorine sources with distinct reactivity profiles. This review aims to give context to these transformations and strategies, highlighting the different tactics to achieve fluorination of benzylic C–H bonds.

## Introduction

**Figure 1:**
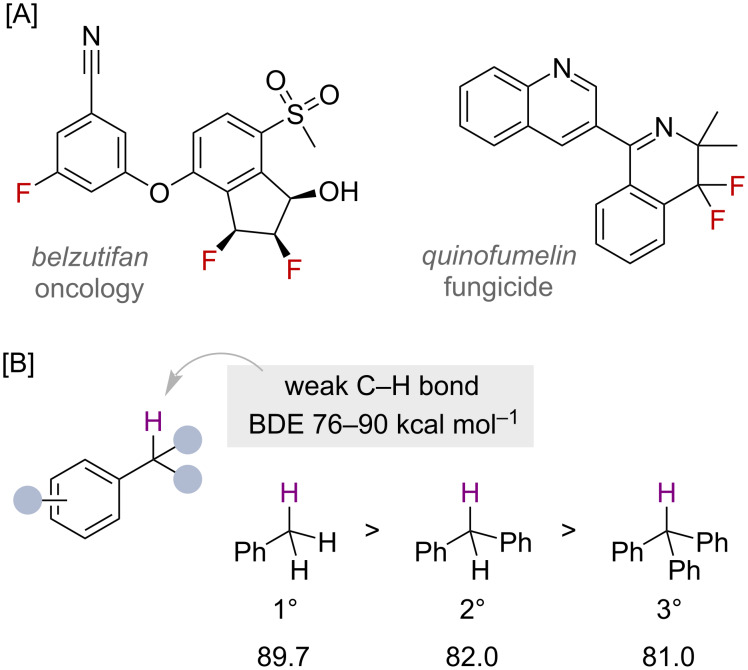
A) Benzylic fluorides in bioactive compounds, with B) the relative BDEs of different benzylic C–H bonds reported in kcal mol^−1^.

The development of new fluorination methodologies is driven largely by the beneficial effects of including fluorine into bioactive molecules. These advantages include the modulation of potency, bioavailability and physical properties of drug and agrochemical compounds [1–3]. The significance of fluorination is reflected in the fact that a large number of agrochemicals contain fluorine, and that almost a quarter of drug molecules approved by the FDA between 2018 and 2022 contained at least one fluorine atom, for example belzutifan and quinofumelin, Figure 1A [4–5].

The fluorination of functionalised carbon centres is a reliable strategy to incorporate fluorine into compounds of interest, with regio and site selectivity pre-determined by the nature of the functionalised carbon. However, the development of C(sp^3^)–H fluorination methods represents a more sustainable and versatile approach, as there is no requirement to pre-functionalise the compound, carry that functional group through synthesis and also protect any potentially labile group that would otherwise displace during the installation of the fluorine atom [6–8]. Therefore, methodologies for the selective C–H fluorination represent a valuable class of reactions [1,9–10], for which several have been disclosed in the chemical literature [11–12].

Benzylic C(sp^3^)–H bonds are comparatively weaker compared to unactivated C(sp^3^)–H bonds, with bond dissociation enthalpies (BDEs) falling in the range of 76–90 kcal mol^−1^ (Figure 1B), due to the increased stability of benzylic radicals and ions imparted through delocalisation with the adjacent π-system [13–15]. In general, the more stabilised the benzylic radical, the weaker the C(sp^3^)–H bond, as demonstrated when considering the BDEs of a series of phenyl-substituted methanes (Figure 1B). The changes in BDE correlate with the relative stability of primary, secondary and tertiary benzylic radicals and cations. As a result, the presence of benzylic C(sp^3^)–H bonds in bioactive molecules can be problematic as they are particularly labile to enzymatic oxidation [16], and hence, their functionalisation has become a strategy to overcome this [17]. For this reason, the fluorination of benzylic C(sp^3^)–H bonds has become particularly important in biologically relevant situations. Benzylic C(sp^3^)–H bonds are also present in a large portion of commercially available building blocks, highlighting the appeal for benzylic C(sp^3^)–H functionalisation reactions in drug-discovery campaigns [17]. Although much is unknown about the precise details, several benzylic fluorides have been reported to be unstable, which is an effect that is apparently dependent on the substitution of the ring. While primary benzylic fluorides are predominately considered to be stable to isolation conditions, secondary and tertiary suffer from the elimination of HF, especially in the presence of silica gel or glass vessels. Therefore, benzyl fluorides have been derivatised, for example in C–O, C–N and C–C bond-forming reactions [18–20], thereby also demonstrating their suitability, as precursors for further functionalisation.

Reviews on the broad area of C–H fluorination have been written [11–12,21–29] with the focus varying, for example between aliphatic fluorination [23], α-fluorination of carbonyl compounds [30], photosensitised C–H fluorination [21,26], recent advances [24] and mechanistic approaches [11]. Examples of specifically benzylic C(sp^3^)–H fluorination reactions are included into many of these reports, as well as in sections of reviews with a much broader scope [12,27–28], and alternative routes to benzylic fluorides have also been reviewed, such as through deoxyfluorination, C–X fluorination, or decarboxylative fluorination [22,31–33]. However, a comprehensive review that focusses specifically on benzylic C–H bonds is still currently missing in the literature. Therefore, we aim to cover reports that focus specifically on benzylic C(sp^3^)–H fluorination, emphasising the most recent protocols but with also some historical context. We also signpost readers to reports where benzylic C–H fluorination has been included, but is not the focus of the work. We have organised the review into different mechanistic strategies, namely, electrophilic, radical and nucleophilic approaches, and highlighted when emerging technologies, such as photo- and electrochemistry effect the desired transformation [22,27].

## Review

### Electrophilic benzylic C(sp^3^)–H fluorination

#### Base mediated

**Figure 2:**
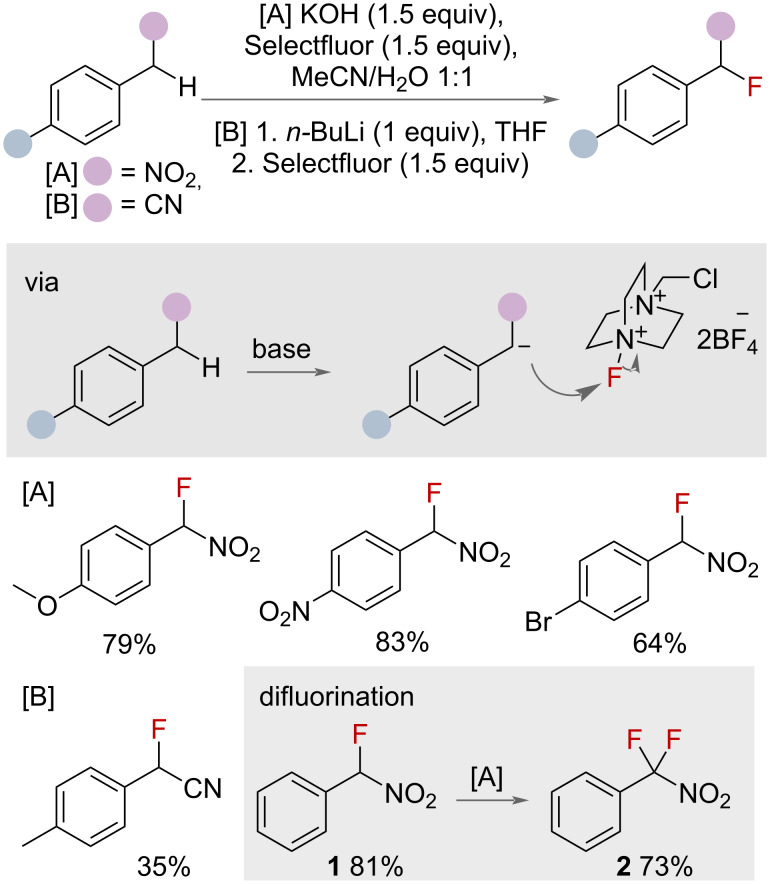
Base-mediated benzylic fluorination with Selectfluor.

Electrophilic fluorinating reagents have been used to effect the transformation of benzylic C(sp^3^)–H to C(sp^3^)–F bonds [22]. Shreeve and co-workers reported the use of KOH or *n-*BuLi to deprotonate acidic protons at benzylic positions adjacent to electron-withdrawing nitro or nitrile groups, respectively, generating benzylic anions that subsequently attack electrophilic Selectfluor to afford the benzyl fluoride (Figure 2) [34]. The methodology was demonstrated on eight *para*-substituted benzylic substrates. The authors noted that resubjecting the monofluorinated compound **1** to the same reaction conditions afforded the difluorinated compound **2** in good yield. The requirement of adjacent to nitro or nitrile groups limits the scope of this approach. Furthermore, the use of strong bases, particularly *n-*BuLi, prevents the application of this methodology on any substrate bearing sensitive functional groups.

**Figure 3:**
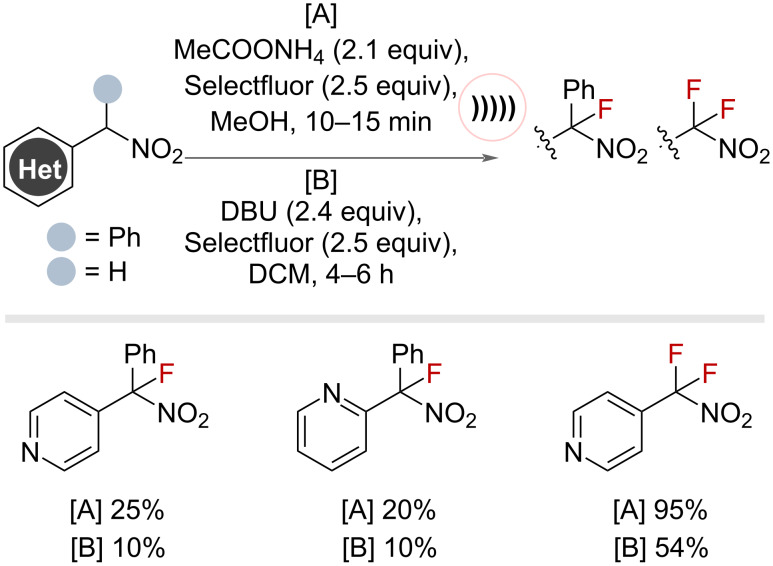
Sonochemical base-mediated benzylic fluorination with Selectfluor.

An analogous method for monofluorination of tertiary benzylic C(sp^3^)–H bonds adjacent to nitro groups was reported by Loghmani-Khouzani and co-workers in 2006, in which ammonium acetate and Selectfluor were employed under sonochemical conditions to effect the fluorination (Figure 3) [35]. The authors noted that the use of sonochemistry afforded higher yields and shorter reaction times compared to standard stirring conditions with DBU. When employing substrates bearing secondary benzylic sites in the reaction conditions, the difluorinated products were observed exclusively in high yields.

**Figure 4:**
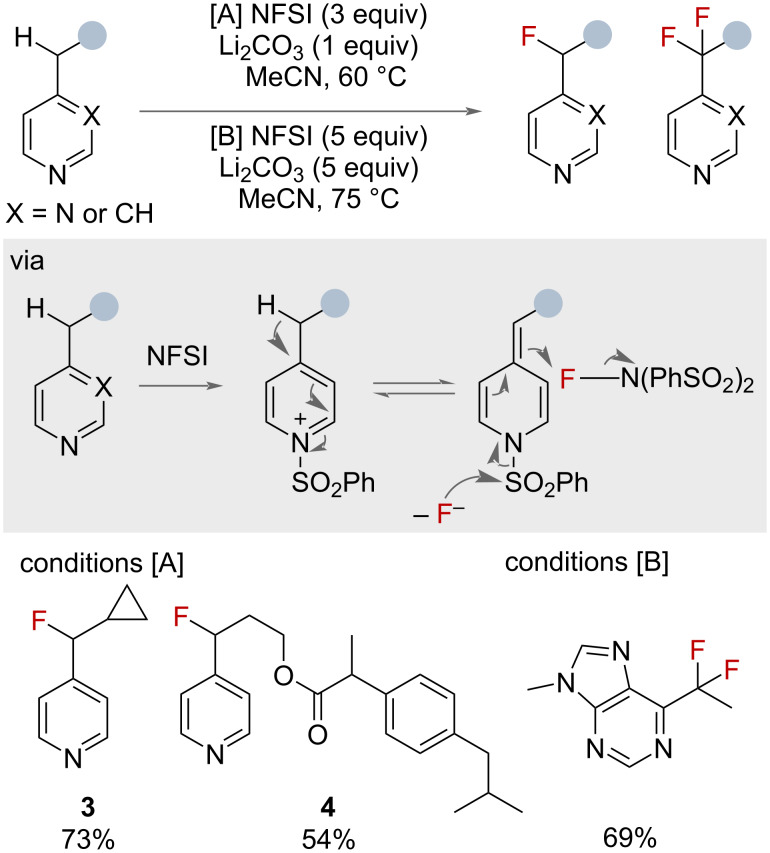
Mono- and difluorination of nitrogen-containing heteroaromatic benzylic substrates.

In 2016, Britton and co-workers reported a method for the efficient monofluorination of 4- and 2-alkylpyridines (Figure 4 – conditions [A]) [36]. The transformation relied on the polarisation of the heterobenzylic C–H bond, via the intermediate formation of an *N*-sulphonylpyridinium salt, to promote deprotonation. Following a polar mechanism with excess NFSI, the heterobenzyl fluoride is obtained. In the case of product **3**, the authors suggested that the absence of radical clock rearrangement products supported a polar mechanism. Conveniently, when both benzylic and heterobenzylic C–H bonds were present in a substrate, the reaction was selective for the heterobenzylic position, as shown by compound **4**. In 2018, a subsequent publication by the same group detailed the use of increased lithium carbonate and NFSI loadings (conditions [B]) to access the difluorinated products [37]. This report also demonstrated a single example of ^18^F monofluorination radiolabelling using [^18^F]NFSI.

Electrophilic fluorination of benzylic C–H bonds has been demonstrated as a powerful approach. However, these techniques can be constrained to defined substrate classes and the requirement of using strong bases.

#### Palladium catalysis

Palladium-catalysed chemistry is pervasive in organic synthesis and can also be used to efficiently fluorinate benzylic C(sp^3^)–H bonds. The general blueprint for this transformation follows a metal insertion into the C(sp^3^)–H bond followed by C–F reductive elimination [11,22,38].

**Figure 5:**
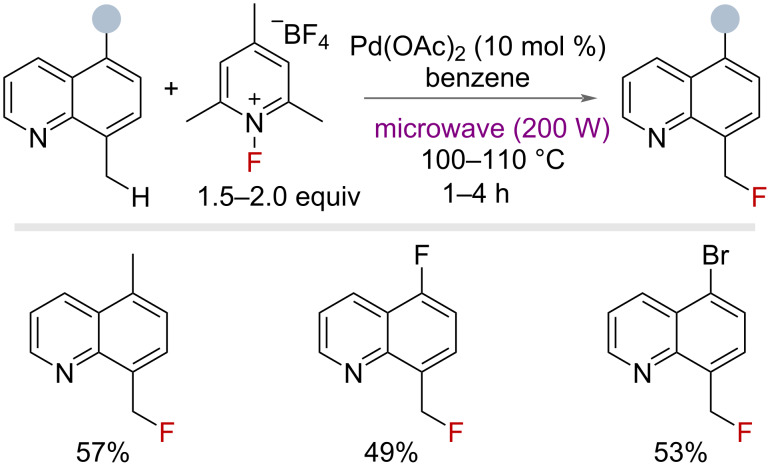
Palladium-catalysed benzylic C–H fluorination with *N*-fluoro-2,4,6-trimethylpyridinium tetrafluoroborate.

In 2006, Sanford and co-workers published a seminal and pioneering report into palladium(II)/(IV)-catalysed C–H fluorination of 8-methylquinolines using *N*-fluoro-2,4,6-trimethylpyridinium tetrafluoroborate as an electrophilic “F^+^” source under microwave conditions (Figure 5) [39]. Benzylic fluorination was achieved in good yields on three examples, each bearing different functional groups at the 5-position.

**Figure 6:**
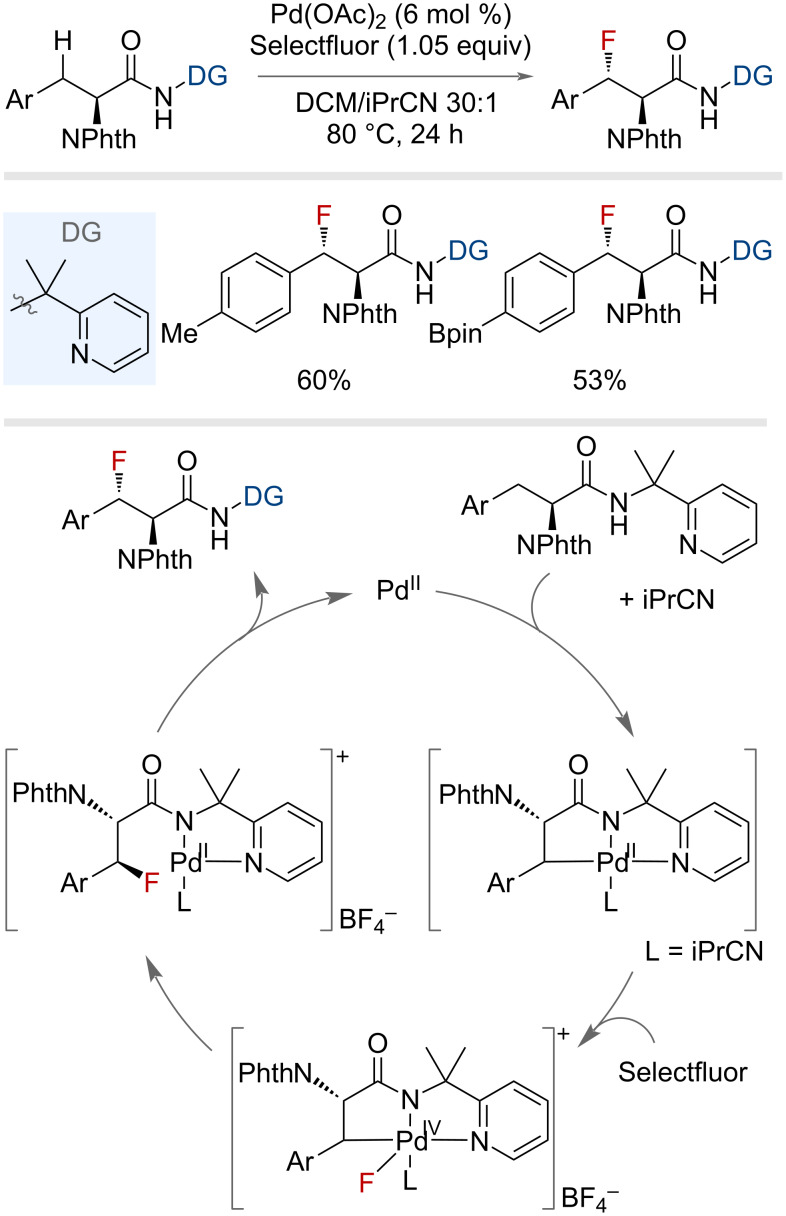
Palladium-catalysed, PIP-directed benzylic C(sp^3^)–H fluorination of α-amino acids and proposed mechanism.

The Shi group reported the use of Pd(II) and Selectfluor to enable the enantioselective β-fluorination of α-amino acids (Figure 6) [40]. The presence of 2-(pyridin-2-yl)isopropylamine (PIP) as directing group was essential for the formation of a four-coordinate palladacycle intermediate, defining the stereochemical outcome. Subsequent oxidation to the Pd(IV)–F species, which triggered reductive elimination, afforded the fluorinated product. The non-innocent behaviour of the isobutyrylnitrile co-solvent aided in stabilising the palladacycle through occupying the vacant coordination site. By installing a cleavable directing group, the authors were able to extend the scope reported by Sanford and co-workers outside of 8-aminoquinoline substrates. Multiple electron-donating and withdrawing groups on the ring were tolerated, including the pinacolborane group; however, the methodology was only shown on secondary benzylic positions.

**Figure 7:**
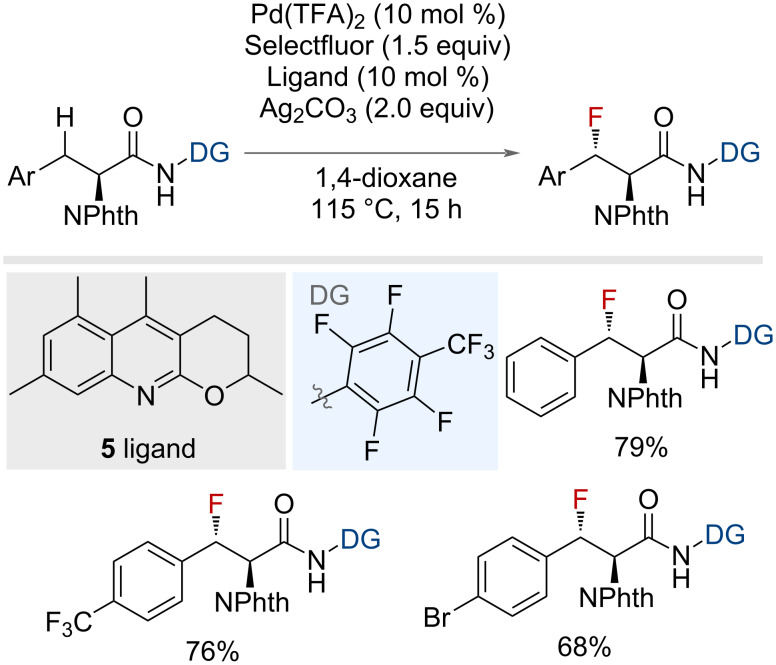
Palladium-catalysed monodentate-directed benzylic C(sp^3^)–H fluorination of α-amino acids.

The stereoselective benzylic monofluorination of α-amino acids was also reported by Yu and co-workers, employing a similar directing group strategy (Figure 7) [41]. The use of the monodentate directing group 2,3,5,6-tetrafluoro-4-(trifluoromethyl)aniline in conjunction with external ligand **5** facilitated the formation of a series of fluorinated α-amino acids.

**Figure 8:**
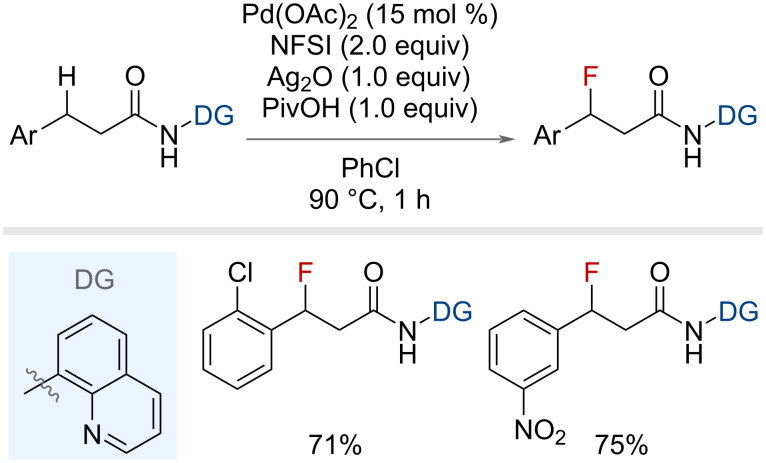
Palladium-catalysed bidentate-directed benzylic C(sp^3^)–H fluorination.

Xu and co-workers also disclosed a palladium-catalysed protocol for the fluorination of simple benzylic substrates bearing a bidentate directing group (Figure 8) [42]. Yields varied from 61–75% across a series of nine benzylic substrates with various substitution patterns on the aromatic ring.

**Figure 9:**
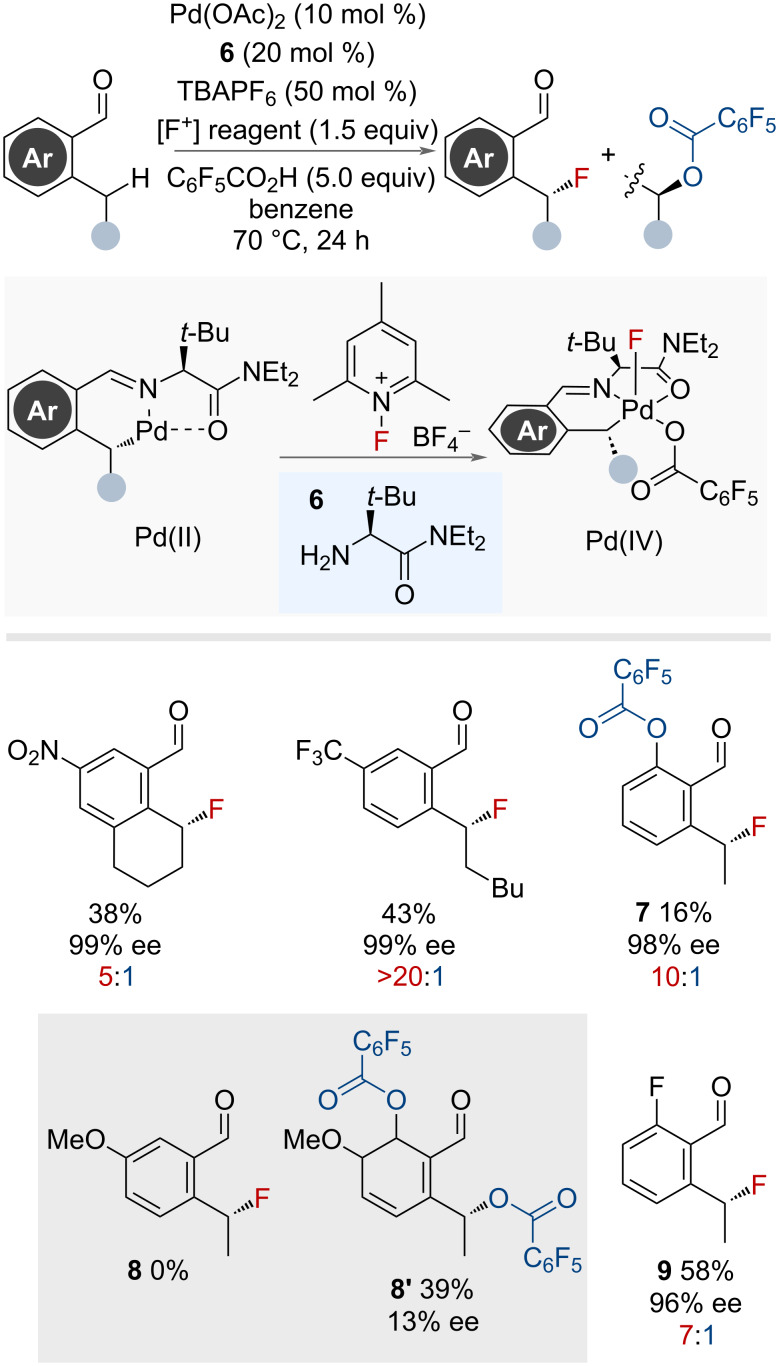
Palladium-catalysed benzylic fluorination using a transient directing group approach. Ratio refers to fluorination (red) vs oxygenation (blue) product.

In 2018, Yu and co-workers reported a palladium-catalysed enantioselective fluorination of benzylic C(sp^3^)–H bonds with the use of a transient chiral directing group **6** [43]. This approach was effective for the stereoselective fluorination of benzylic positions *ortho* to aldehyde substituents (Figure 9). The choice of a bulky amino, transient, directing group dictated the stereochemical outcome and promoted the C–F reductive elimination through an inner-sphere pathway. A competitive C–O bond formation to afford the acyloxylation product was observed, and favoured when using directing groups with less steric bulk. This product had the opposite stereochemistry to the fluorination product suggesting it occurred via a competitive S_N_2 pathway. This is supported by the selectivity for C–O bond formation for substrates bearing primary benzylic positions, attributed to the faster rate of S_N_2 at the less hindered carbon. The scope was limited to substrates bearing secondary benzylic sites, with various functional groups tolerated. However, substrates bearing electron-donating substituents on the arene were unsuccessful. Without substituents on the ring, aryl C–H activation and subsequent C–O bond formation occurred along with benzylic fluorination (**7**) (low efficiency). The presence of a *p*-methoxy group resulted in a switch in selectivity to acyloxylation **8’** as the major product. The authors displayed the stability of the secondary benzyl fluoride **9** to various S_N_Ar conditions.

While these methods demonstrate excellent application of palladium catalysts to perform benzylic fluorinations, the need to install a directing group can limit substrate scope. Therefore, methods that can achieve the same transformation in the absence of a directing group are particularly attractive.

### Radical benzylic C(sp^3^)–H fluorination

**Figure 10:**
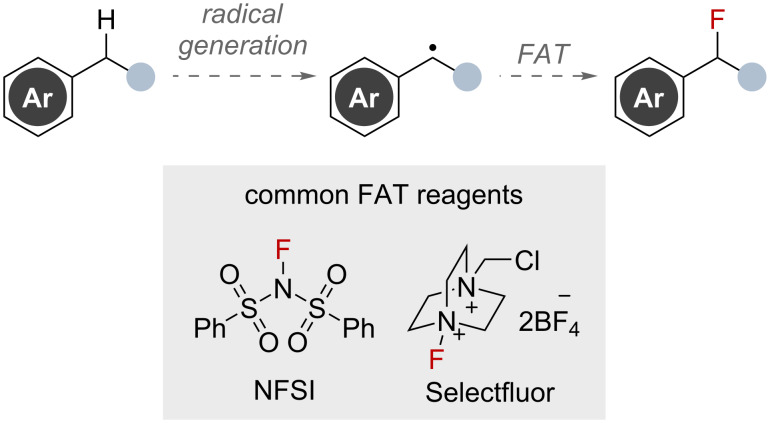
Outline for benzylic C(sp^3^)–H fluorination via radical intermediates.

Radical fluorination techniques are an attractive approach for benzylic C–H fluorinations that are shown to proceed without a directing group. Carbon-centred radical generation at the benzylic position is known to occur via multiple pathways [44–47]. These radicals can then undergo fluorination via fluorine-atom-transfer (FAT) with various reagents capable of SET pathways, such as Selectfluor and NFSI (Figure 10) [48]. By avoiding the need for strong bases and directing group strategies, this approach opens the door to fluorinating a wider range of benzylic substrates.

#### Metal catalysed

**Figure 11:**
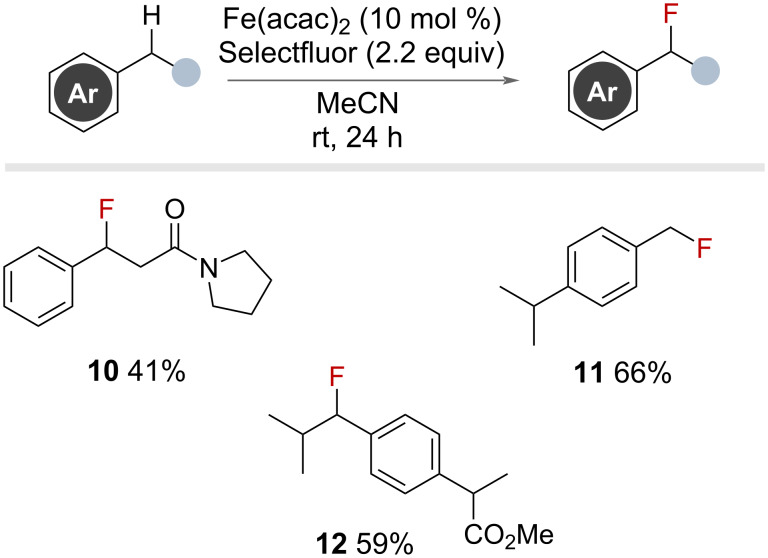
Iron(II)-catalysed radical benzylic C(sp^3^)–H fluorination using Selectfluor.

In 2013, Lectka and co-workers reported an iron(II)-catalysed benzylic fluorination with Selectfluor (Figure 11) [49]. The authors were able to use an inexpensive iron source to promote the fluorination of a range of primary and secondary benzylic substrates that were not too electron-rich nor too electron-poor. Interestingly, selectivity for the benzylic position was observed over α-halogenation in substrates bearing carbonyl groups (41% yield for **10**). The conditions were selective for primary benzylic fluorination (**11**) and secondary benzylic fluorination (**12**) in the presence of tertiary benzylic sites. Although no mechanism has been proposed, the authors concluded it likely proceeded via a radical pathway [23].

**Figure 12:**
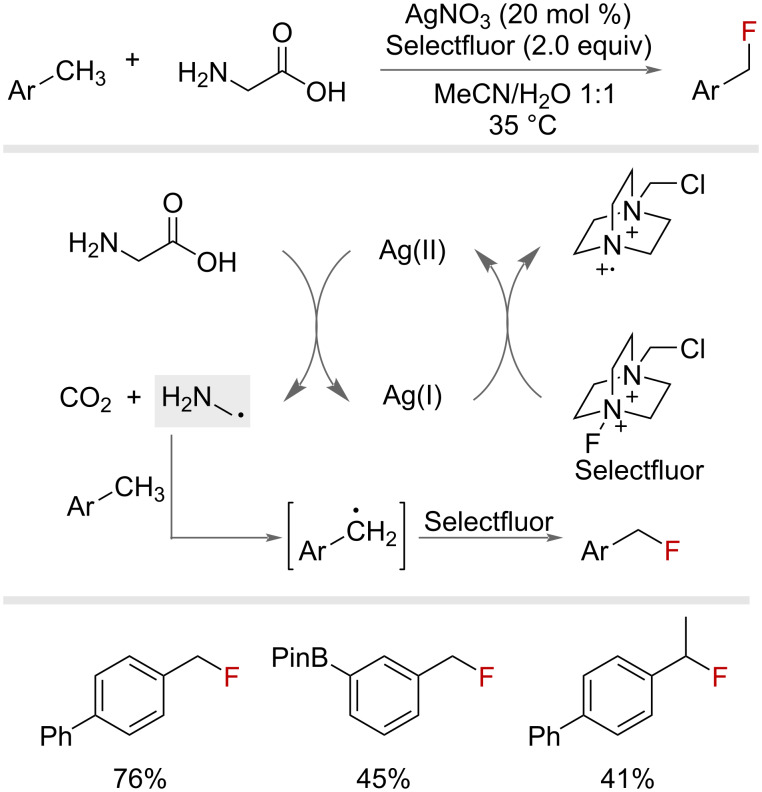
Silver and amino acid-mediated benzylic fluorination.

In 2017, Baxter and co-workers introduced a silver-catalysed benzylic fluorination method that employed unprotected amino acids as radical precursors, Figure 12 [50]. Oxidation of glycine by Ag(II) promotes decarboxylation and results in the α-amino radical, which performs a HAT on the benzylic substrate to furnish the benzylic radical. This subsequently undergoes FAT with Selectfluor to produce the desired benzyl fluoride. Increasing amino acid and Selectfluor loadings achieved difluorination of the benzylic substrates. This procedure was demonstrated predominately on primary benzylic substrates, but could be used to effect the fluorination of several secondary and tertiary substrates too.

**Figure 13:**
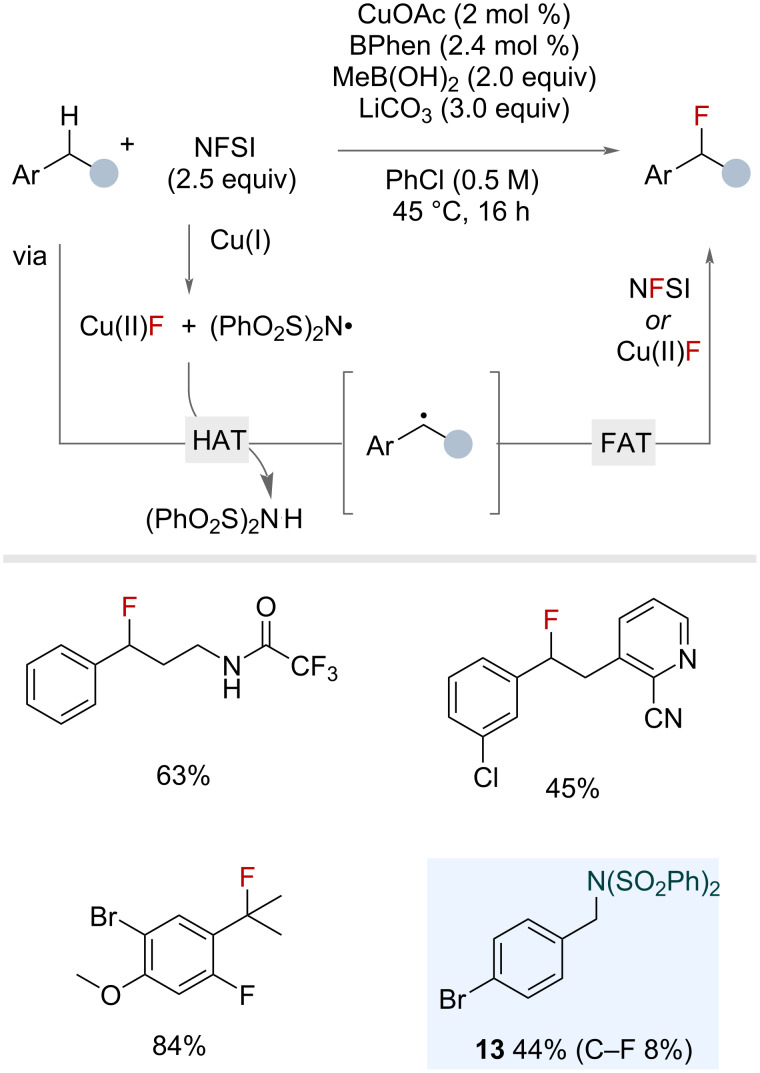
Copper-catalysed radical benzylic C(sp^3^)–H fluorination using NFSI.

In 2012, Lectka reported a fluorination of mostly aliphatic C–H bonds that used a molecularly defined copper catalyst with a bis imine ligand, along with co-catalytic *N*-hydroxyphthalimide and a phase-transfer catalyst [51]. Although only a few benzylic substrates were shown, this report provided important precedent for the ability of copper fluoride species to deliver fluorine to carbon radicals. Following on from this, Stahl and co-workers reported in 2020 an efficient synthesis of secondary and tertiary benzyl fluorides via a copper-catalysed radical relay mechanism. Excess NFSI functioned as both a fluorine source and HAT reagent precursor (Figure 13) [20]. Fluorine abstraction from NFSI by copper(I) generates an *N*-centred radical that is selective for benzylic C(sp^3^)–H bonds [52–53], affording the benzylic radical via HAT. Subsequent FAT with the in situ-generated Cu(II)F or NFSI affords the benzyl fluoride. Substrates bearing secondary and tertiary benzylic sites were successful in the reaction. However, primary benzylic substrates were not tolerated, instead affording the N(SO_2_Ph)_2_ adduct (e.g., product **13**) in moderate yields. The authors noted that several secondary and tertiary benzyl fluorides were unstable to silica during isolation or storage in glass vessels, and therefore, demonstrated several downstream diversifications of the benzyl fluorides.

**Figure 14:**
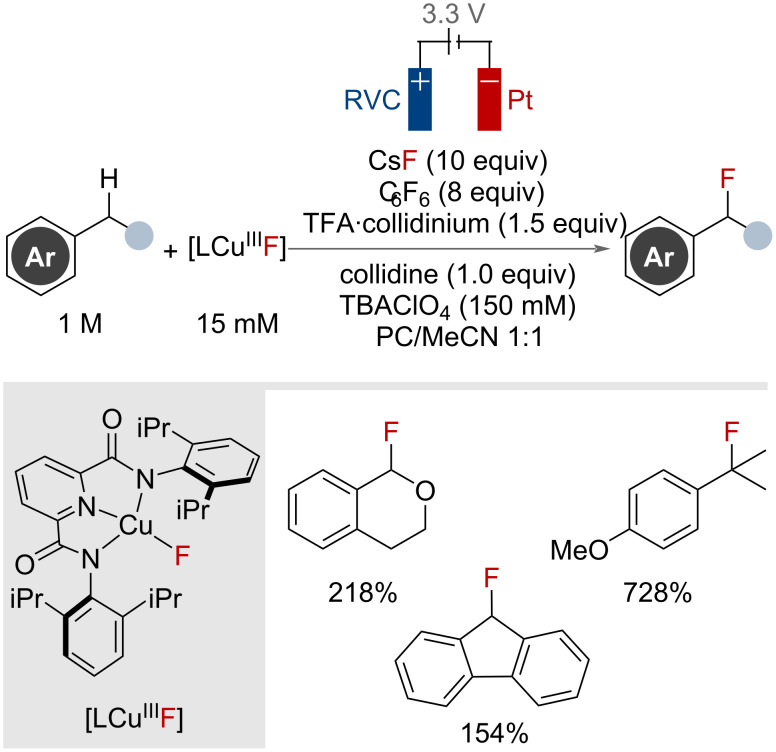
Copper-catalysed C(sp^3^)–H fluorination of benzylic substrates with electrochemical catalyst regeneration**.** Yields are NMR yields quoted vs copper catalyst.

Sevov, Zhang and co-workers reported in 2023 a stable copper(III) fluoride complex that was capable of C(sp^3^)–H activation and fluorination, including on one tertiary and five secondary benzylic substrates (Figure 14) [54]. This work utilised electrochemical oxidation with a nucleophilic source of fluoride, CsF, to regenerate the trisligated copper(III) fluoride complex.

**Figure 15:**
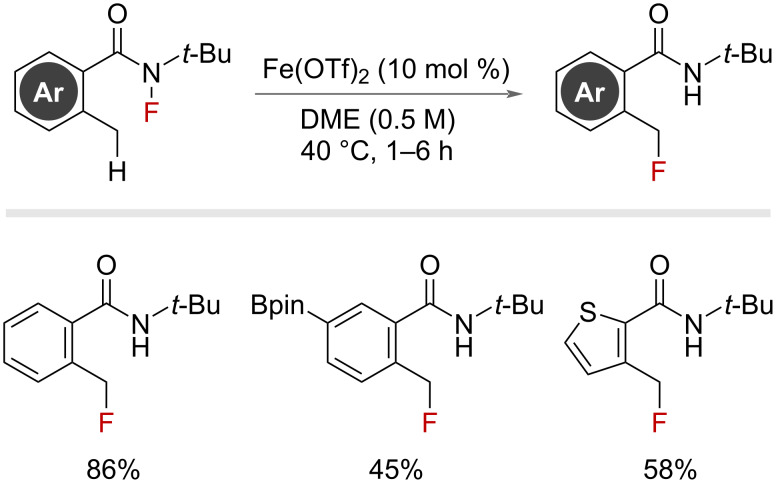
Iron-catalysed intramolecular fluorine-atom-transfer from N–F amides.

In 2016, Silas reported an intramolecular fluorine-atom-transfer (FAT) from an *N*-fluorinated amide to a pendant carbon-based radical formed from an iron catalyst (Figure 15) [55–56]. This concept of fluorine transfer through a 6-membered transition state was shown to work efficiently from primary, as well as secondary, benzylic radicals that have an *ortho*-substituted *tert*-butylamide moiety.

**Figure 16:**
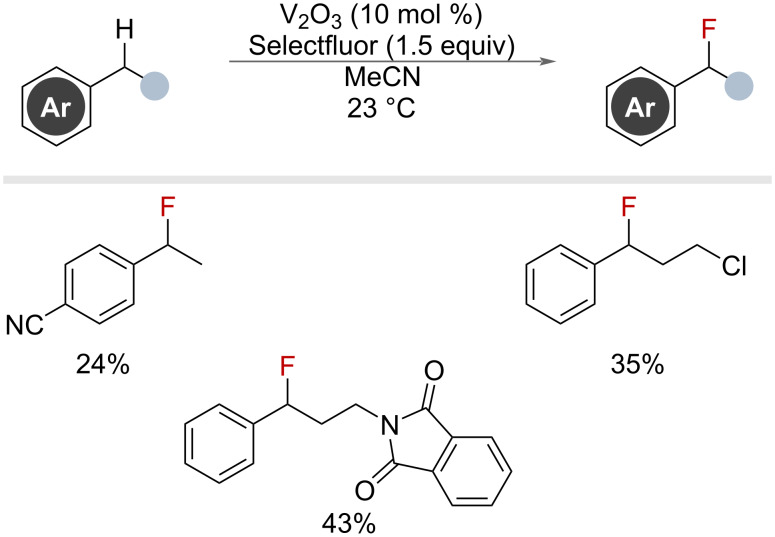
Vanadium-catalysed benzylic fluorination with Selectfluor.

Finally, while not focussing on benzylic substrates, a vanadium-mediated fluorination of aliphatic C–H bonds was reported by Chen and co-workers, which also included five benzylic substrates (Figure 16) [57].

#### Metal free

**Figure 17:**
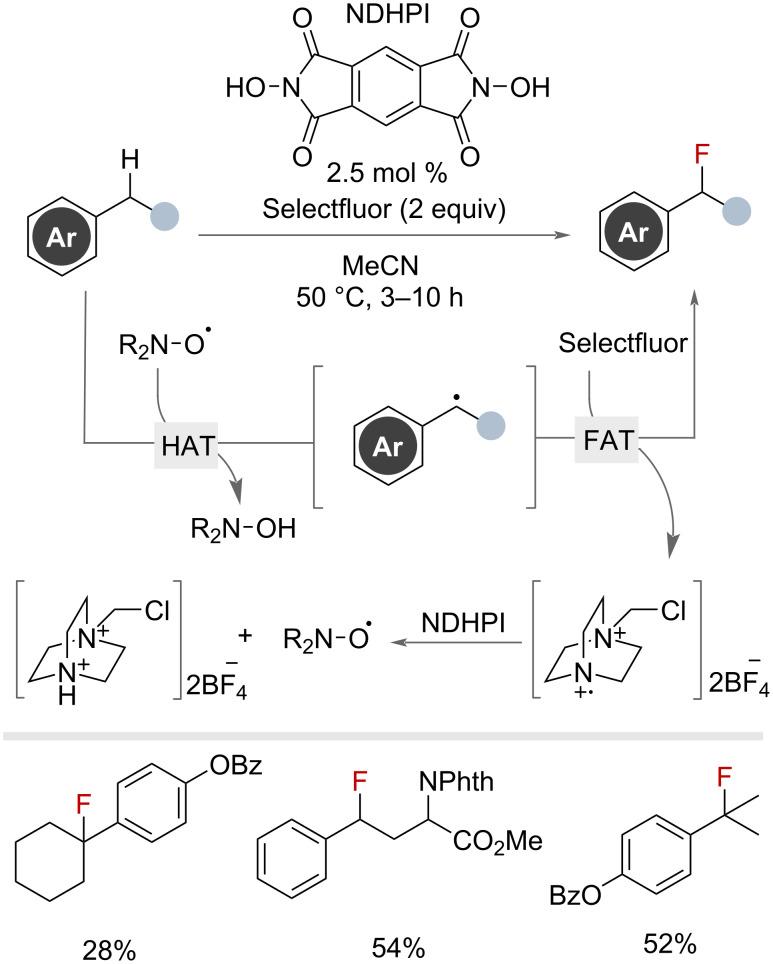
NDHPI-catalysed radical benzylic C(sp^3^)–H fluorination with Selectfluor.

Numerous reports have detailed metal-free radical C(sp^3^)–H fluorinations suitable for benzylic substrates. These typically involve the generation of a HAT reagent that is selective for benzylic C–H bonds and facilitates the generation of a benzylic radical. Subsequent FAT, from a fluorinating reagent, yields the desired benzyl fluorides. In 2013, Inoue and co-workers demonstrated the use of catalytic *N*,*N*-dihydroxypyromellitimide (NDHPI) as a precursor for *N*-oxyl radicals that serve as the HAT reagent. Selectfluor was employed as the FAT reagent, generating an *N*-centred radical on the spent Selectfluor that can regenerate the *N*-oxyl radicals from NDHPI (Figure 17) [58]. The secondary and tertiary substrates selected were shown to undergo this transformation in moderate to good yields.

**Figure 18:**
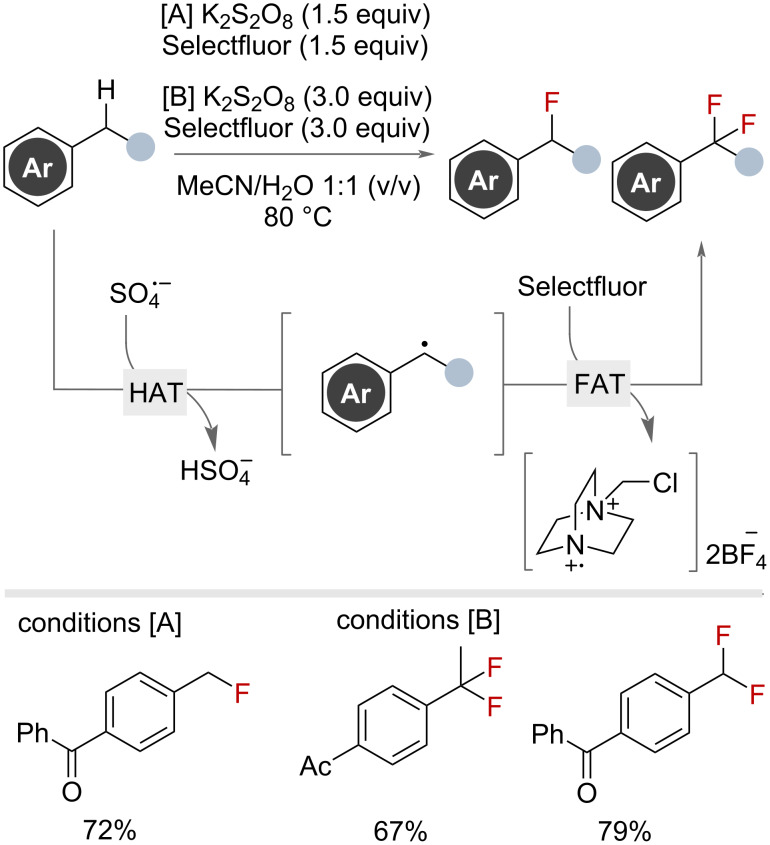
Potassium persulfate-mediated radical benzylic C(sp^3^)–H fluorination with Selectfluor.

The Yi group published a complementary method using stoichiometric potassium persulfate as the HAT reagent precursor (Figure 18) [59]. The authors proposed that under heating K_2_S_2_O_8_ decomposed to SO_4_^•−^ which could then abstract the benzylic hydrogen to generate the benzylic radical. Fluorine-atom-transfer with Selectfluor then afforded the benzyl fluoride. Other fluorinating reagents such as NFSI or DAST did not perform as well. By varying the loadings of K_2_S_2_O_8_ and Selectfluor, selectivity for the mono- (conditions A) or difluorination (conditions B) products could be achieved.

**Figure 19:**
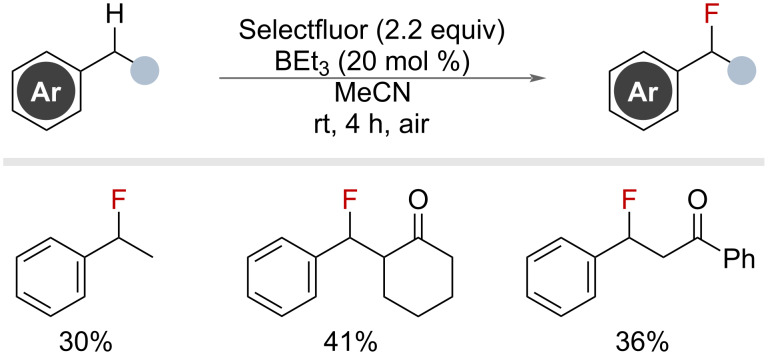
Benzylic fluorination using triethylborane as a radical chain initiator.

Building on their previous iron-catalysed work, Figure 11, Lectka and co-workers reported in 2014 the use of triethylborane as a radical chain initiator for C(sp^3^)–H fluorination. They demonstrated this reaction primarily on alkyl substrates, but 5 secondary benzylic substrates were also shown to undergo the reaction effectively (Figure 19) [60]. The authors proposed the transformation occurred via established triethylborane autoxidation initiation and propagation methods, noting the importance of high purity reagents and the presence of O_2_.

**Figure 20:**
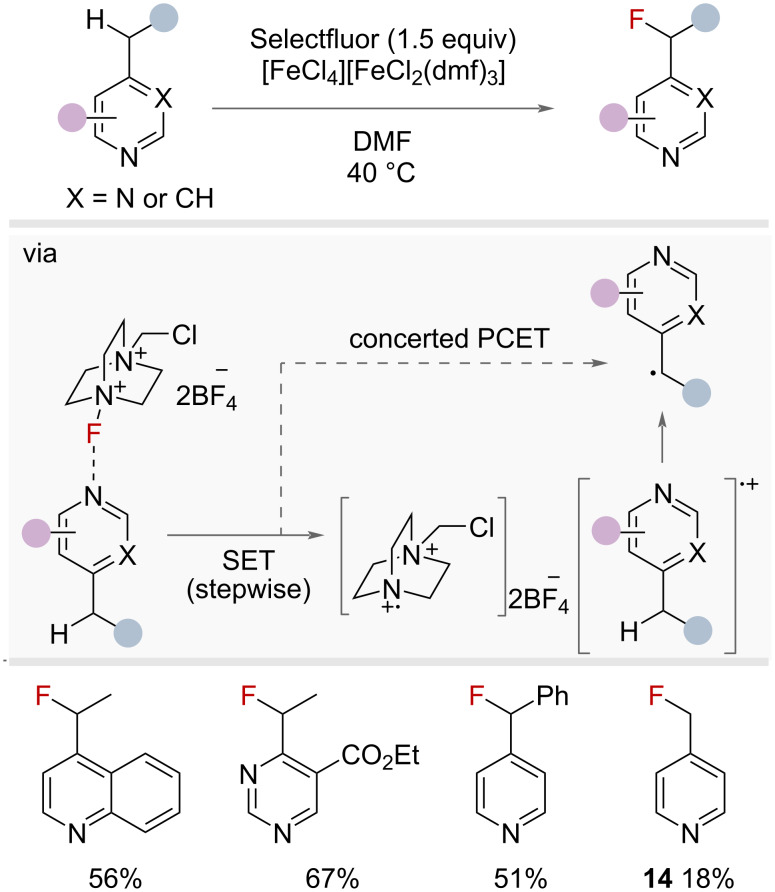
Heterobenzylic C(sp^3^)–H radical fluorination with Selectfluor.

Radical fluorination of hetereobenzylic C(sp^3^)–H bonds was demonstrated by Van Humbeck and co-workers in 2018, who enabled the fluorination of aza-heterocycles at the benzylic position using Selectfluor (Figure 20) [61]. The authors proposed the formation of a charge-transfer complex between the heterocycle and Selectfluor, capable of promoting an ET/PT or PCET pathway to furnish the carbon-centred radical at the heterobenzylic position. Fluorine-atom-transfer with Selectfluor then afforded the desired product. Secondary and tertiary substrates worked well under the reaction conditions, whereas primary positions afforded low yields (**14**). No additive was required to achieve the desired selectivity, but in some cases the addition of small amounts of iron salt [FeCl_4_][FeCl_2_(dmf)_3_] improved yields.

**Figure 21:**
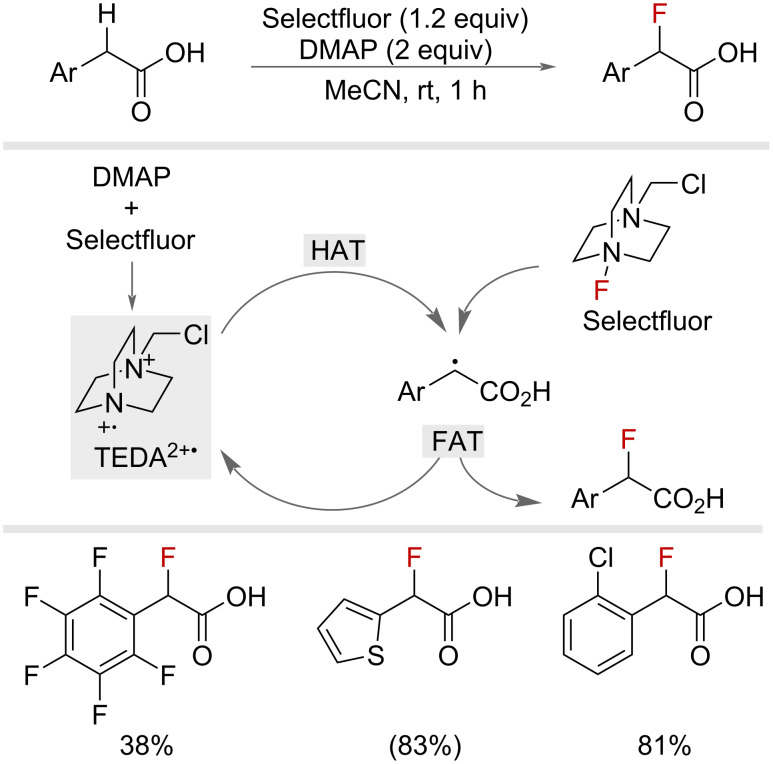
Benzylic fluorination of phenylacetic acids via a charge-transfer complex. NMR yields in parentheses.

In 2022, Pieber and co-workers reported a benzylic fluorination of phenylacetic acids via a charge-transfer complex (Figure 21) [62]. The authors proposed that the combination of Selectfluor and DMAP spontaneously produced the Selectfluor radical dication (TEDA^2+•)^, which served as a radical chain carrier capable of facilitating HAT to produce a benzylic radical. Fluorine-atom-transfer (FAT) with Selectfluor then gave the benzyl fluoride. The low acidity of phenylacetic acids in polar aprotic solvents disfavoured decarboxylation (via an SET pathway) promoting HAT from the benzylic position. By using a mixture of 1:1 MeCN/H_2_O and heating, the decarboxylation pathway could be enabled to afford primary benzyl fluorides.

In the same year, Barham and co-workers also showed that the radical dication TEDA^2+•^ was capable of HAT on unactivated C(sp^3^)–H, enabling fluorination at these positions [63]. This work utilised *para*-fluorobenzoates as both photocatalysts or photo-auxiliaries and was demonstrated on a number of benzylic examples.

#### Photochemical

**Figure 22:**
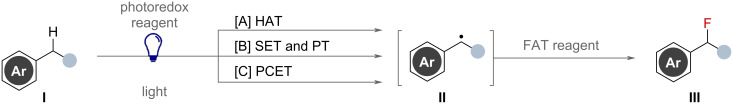
Oxidative radical photochemical benzylic C(sp^3^)–H strategies.

Photochemical methods have proven to be powerful tools in the generation of reactive intermediates, including benzylic radicals [64–67]. Oxidative photochemical functionalisation of benzylic C–H bonds to benzylic radicals can be envisaged to occur through three different pathways (Figure 22). Upon excitation by light, photoredox reagents can induce a number of changes in benzylic substrate **I**, either directly or via mediated processes. Hydrogen-atom-transfer (HAT) results in the concerted transfer of an electron and a proton from the benzylic substrate resulting in the benzylic radical **II** – pathway [A] [67]. This radical can also be accessed via sequential oxidative single-electron-transfer (SET) and proton-transfer (PT) steps (pathway [B]), or concerted proton-coupled electron transfer (PCET) (pathway [C]). Benzylic radicals can then react with FAT reagents to give the desired benzyl fluoride products [66,68].

**Figure 23:**
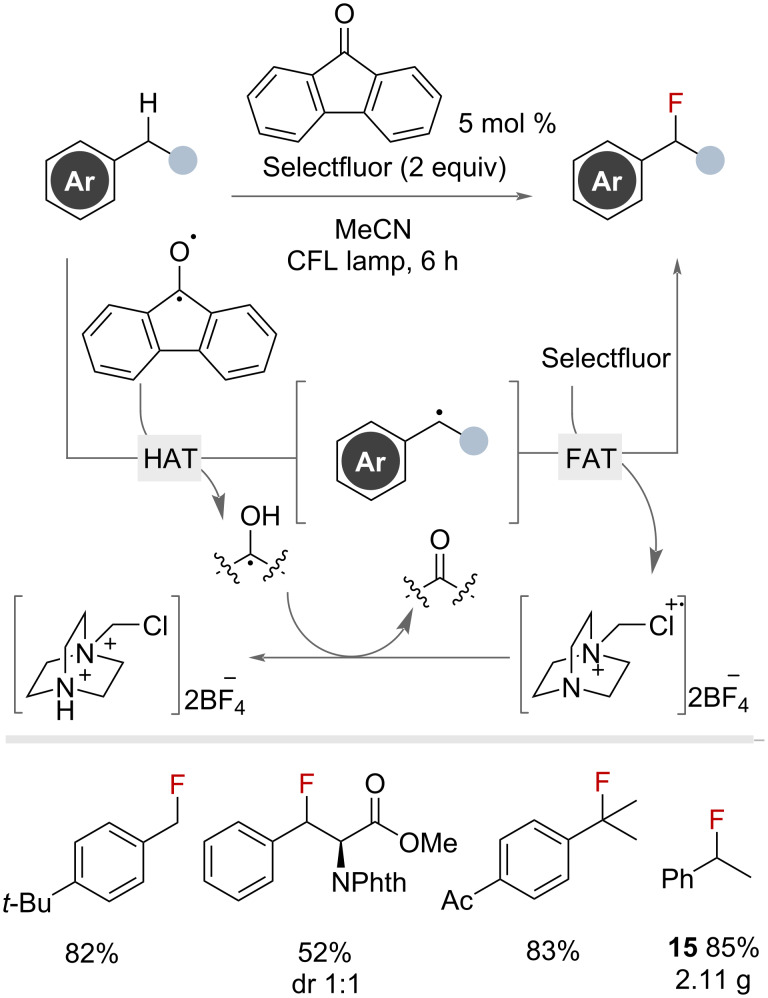
9-Fluorenone-catalysed photochemical radical benzylic fluorination with Selectfluor.

Several photochemical benzylic fluorination methodologies proposed to proceed via radical pathways have been reported. Chen and co-workers published a pioneering report in 2013 that used photocatalyst 9-fluorenone under visible-light irradiation to generate a photoexcited aryl ketone, capable of HAT to promote benzylic fluorination with Selectfluor (Figure 23) [69]. The reaction tolerated an exceptional range of functional groups and enabled the fluorination of primary, secondary and tertiary benzylic substrates. The methodology was amenable to scale up, demonstrating the gram-scale synthesis of product **15** in 85% yield.

**Figure 24:**
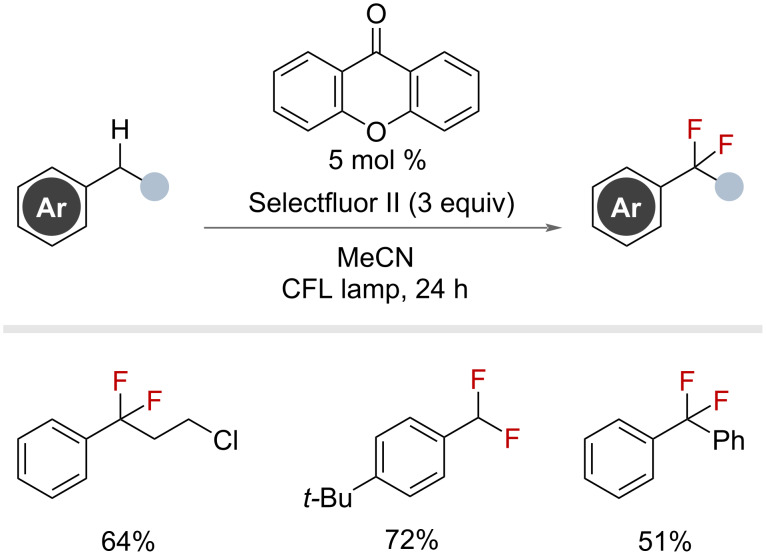
Xanthone-photocatalysed radical benzylic fluorination with Selectfluor II.

The authors recognised the difficulty in sequential fluorination and noted that the use of a more electron-rich photocatalyst would be required to promote hydrogen abstraction. By changing the photocatalyst to xanthone and replacing Selectfluor with 3 equivalents of Selectfluor II, the authors afforded *gem*-difluoride products of primary and secondary benzylic substrates in high yields (Figure 24).

**Figure 25:**
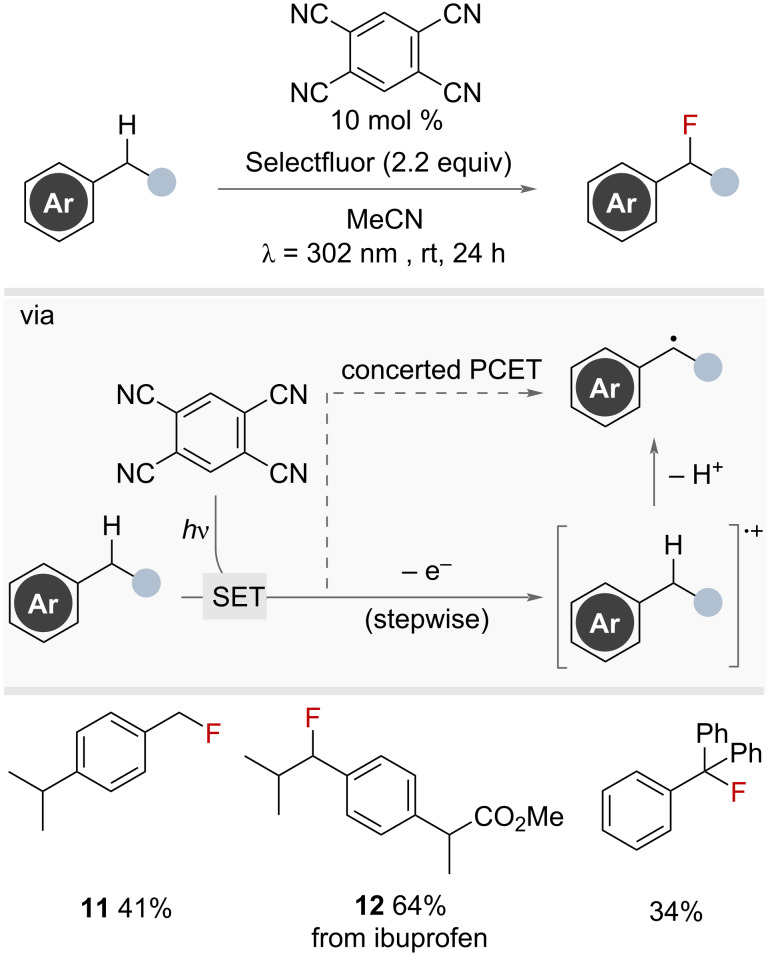
1,2,4,5-Tetracyanobenzene-photocatalysed radical benzylic fluorination with Selectfluor.

In 2014, Lectka and co-workers showed that 1,2,4,5-tetracyanobenzene could be used under ultraviolet light irradiation as a photocatalyst in the fluorination of benzylic C(sp^3^)–H bonds (Figure 25) [70]. Selectfluor was used as the FAT reagent to furnish a selection of primary, secondary and tertiary benzyl fluorides with different functional groups on the aromatic ring and adjacent to the benzylic position. Mechanistic investigations suggested an initial electron transfer to generate a radical cation en route to the intermediate benzylic radical, rather than a HAT process, however, the authors did not distinguish between a stepwise SET and subsequent PT or concerted PCET mechanism. The yields observed using this approach were broadly similar to the same group’s iron-catalysed method (Figure 11).

**Figure 26:**
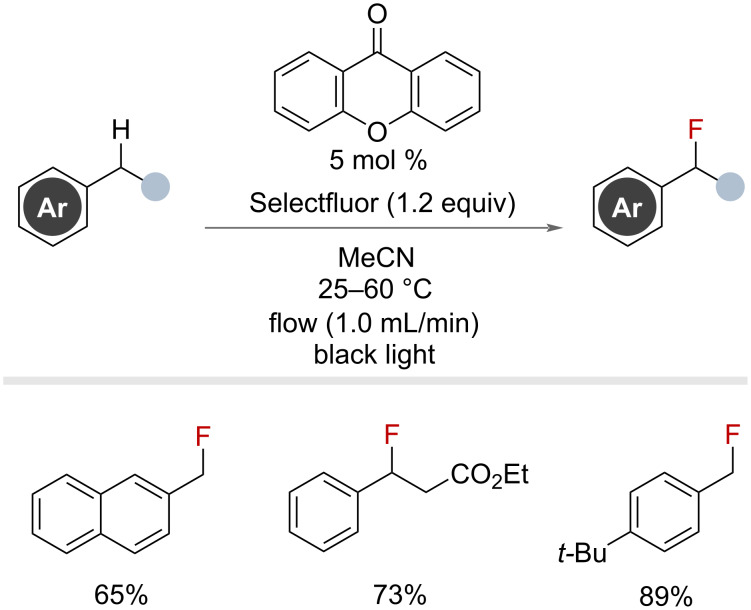
Xanthone-catalysed benzylic fluorination in continuous flow.

In the same year, Cantillo, de Frutos, Kappe and co-workers reported a similar approach, using xanthone as their photocatalyst in a continuous flow system (Figure 26) [71]. The authors were able to demonstrate rapid benzylic fluorination of 13 substrates, requiring residence times below 30 min.

**Figure 27:**
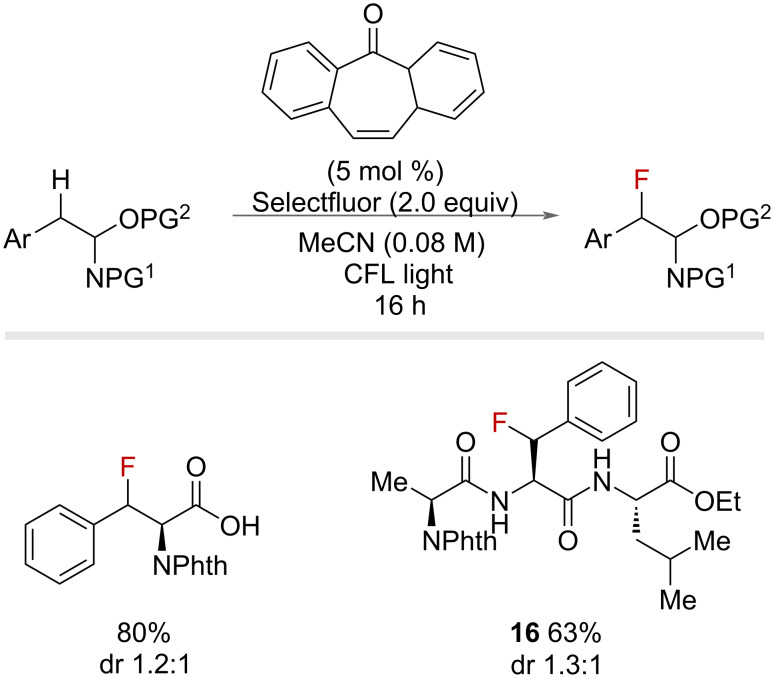
Photochemical phenylalanine fluorination in peptides.

The use of photoexcited aryl ketones was further expanded in 2016 by Lectka and co-workers who reported the use of 5-dibenzosuberenone as a photosensitive arylketone catalyst in the fluorination of phenylalanine residues in peptides (Figure 27) [72]. This work demonstrated high yields and selectivity for peptides bearing phenylalanine residues, including tripeptides, such as **16**.

**Figure 28:**
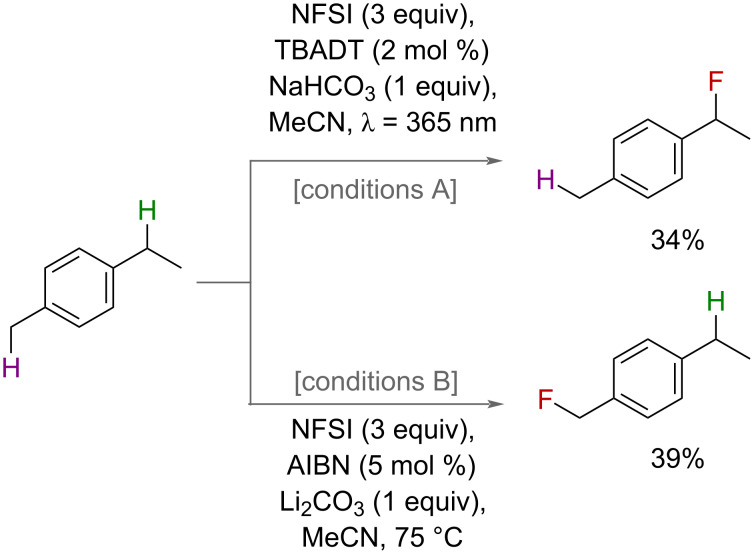
Decatungstate-photocatalyzed versus AIBN-initiated selective benzylic fluorination.

In 2015, Britton and co-workers reported a photochemical HAT-guided approach using NFSI as their fluorine source [73]. The authors demonstrated the use of a decatungstate photocatalyst as a species capable of hydrogen-atom abstraction and use it to access a range of secondary and tertiary benzyl fluorides in moderate to excellent yields. AIBN was also demonstrated as a suitable radical initiator for this transformation, albeit in reduced yields. Interestingly, for substrates bearing both primary and secondary benzylic C(sp^3^)–H bonds, AIBN exhibited selectivity for the primary position and the opposite was seen for the decatungstate catalyst (Figure 28). The authors attributed this to the increased solubility and concentration of NFSI in the AIBN conditions, which were performed at elevated temperatures, promoting facile trapping of a primary radical. In contrast, the decatungstate conditions, which operated at room temperature where NFSI is not completely dissolved and is therefore not as concentrated in solution, allows for equilibration between benzylic radicals towards the more stable secondary radical. This switch in selectivity provides an interesting tool for selective fluorination in substrates with multiple benzylic sites.

**Figure 29:**
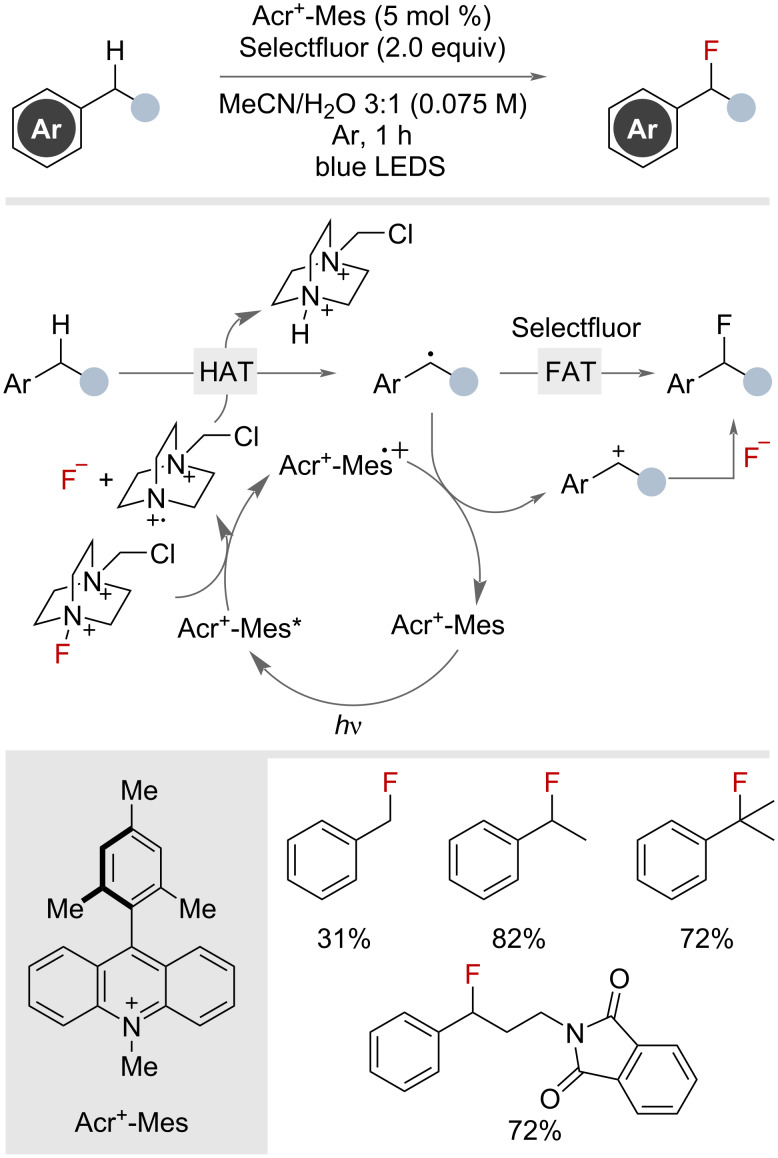
Benzylic fluorination using organic dye Acr^+^-Mes and Selectfluor.

In 2017, Wu and co-workers disclosed the use of catalytic amounts of the organic dye Acr^+^-Mes under visible-light irradiation in combination with stoichiometric amounts of Selectfluor to achieve benzylic fluorination (Figure 29) [74]. It was proposed that a SET between Selectfluor and the photoexcited catalyst liberated fluoride and a potent HAT reagent capable of generating the benzylic radical, which then performs FAT with Selectfluor to generate the desired benzyl fluoride. Alternatively, the benzylic radical could further be oxidized to the cation, and in the process, regenerating the ground-state catalyst. The benzylic cation would then be trapped by the previously liberated fluoride. This reactivity was demonstrated on one primary, one tertiary and eight secondary substrates. When diphenylmethane substrates were subjected to the reaction conditions benzylic ketone products were observed.

As highlighted by the examples in this section, radical-based approaches enable the fluorination of a diverse range of benzylic substrates, which rely on the use of FAT reagents, such as Selectfluor, NFSI or copper fluoride complexes.

### Nucleophilic benzylic C(sp^3^)–H fluorination

Nucleophilic fluorine sources can be more economical from financial and waste perspectives when compared to reagents such as Selectfluor and NFSI [75–77]. This type of fluorine source is also preferred for positron emission tomography (PET) imaging with [^18^F]fluoride [78]. Despite the challenges associated with nucleophilic fluoride, including solubility issues of metal fluoride salts, safety issues with hydrogen fluoride, poor nucleophilicity [79], and side reactivity as a base [75,79], a few elegant examples of nucleophilic benzylic C(sp^3^)–H fluorination have been reported.

#### Metal catalysis

**Figure 30:**
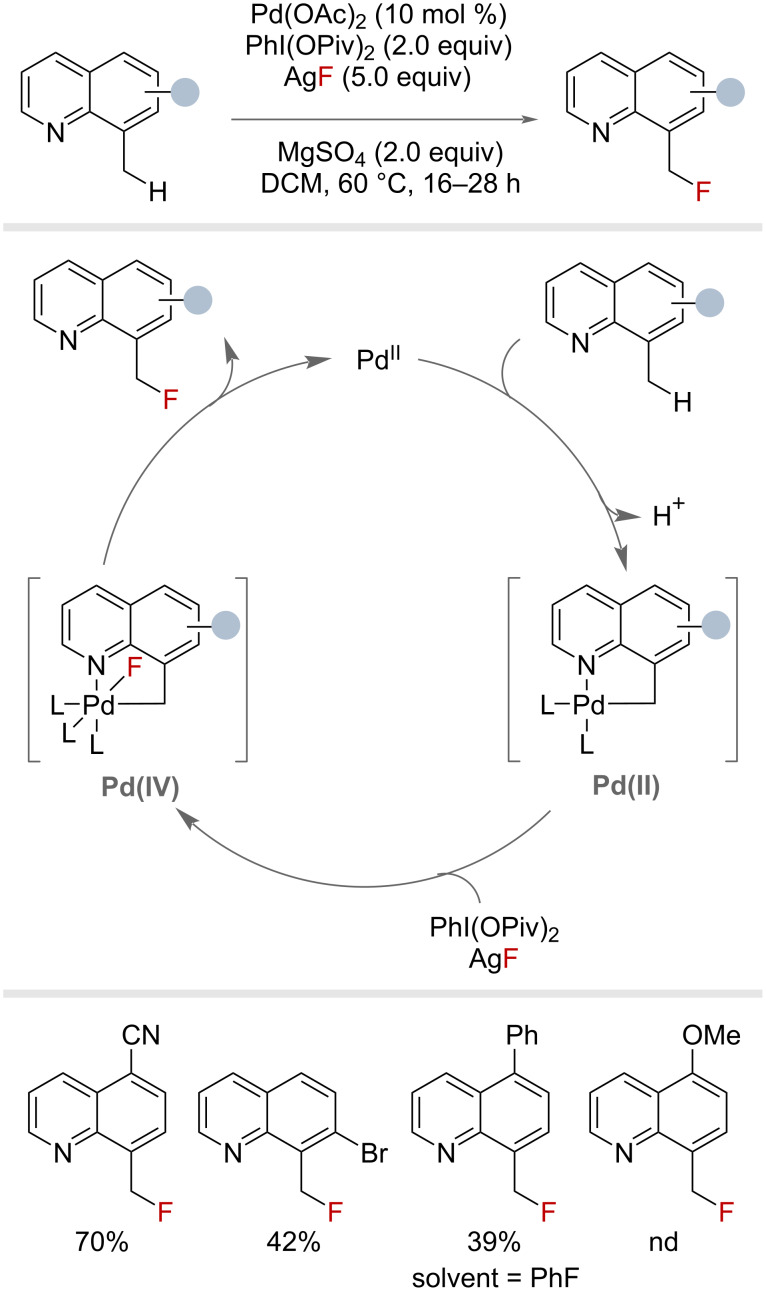
Palladium-catalysed benzylic C(sp^3^)–H fluorination with nucleophilic fluoride.

Fluoride sources have been used in combination with transition-metal complexes to generate metal–fluorine bonds capable of FAT to benzylic substrates. In a follow-up to their work using electrophilic fluorine sources for palladium-catalysed benzylic C–H fluorination (Figure 5), the Sanford group demonstrated in 2012 the same transformation could be achieved with nucleophilic fluoride sources too (Figure 30) [77]. This process involved an initial quinoline-directed C–H activation by Pd(II), followed by oxidation to generate a Pd(IV)–fluoride complex capable of C–F reductive elimination to generate the primary benzyl fluoride. Under this protocol, eleven 8-methylquinoline derivatives could be fluorinated in yields of up to 70%.

**Figure 31:**
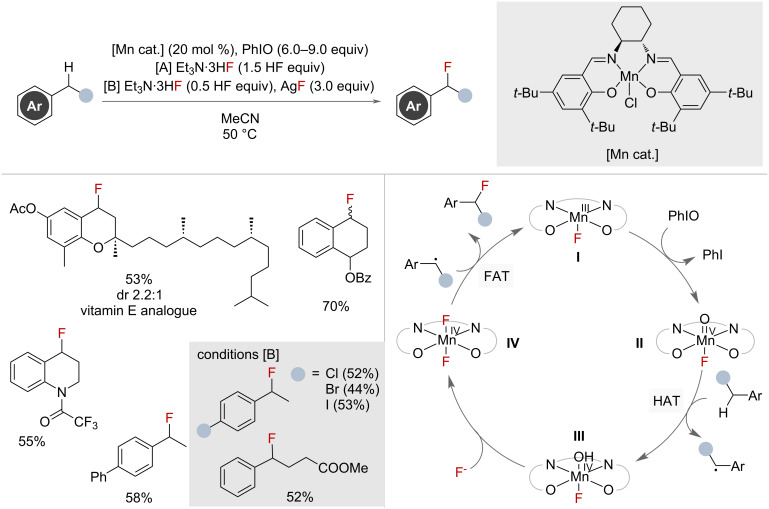
Manganese-catalysed benzylic C(sp^3^)–H fluorination with AgF and Et_3_N·3HF and proposed mechanism. ^19^F NMR yields in parentheses.

In 2013, Groves and co-workers reported the use of manganese salen and manganese porphyrin catalysts in the preparation of a range of secondary benzyl fluorides via C–H fluorination (Figure 31) [80]. Substrates bearing electron-withdrawing substituents on the aryl group benefitted from fewer HF equivalents and the addition of silver fluoride. A follow-up report showed that only minor alterations to the conditions were needed to make the process amenable to the use of [^18^F]KF, facilitating radiofluorination [81]. Both reports used hypervalent iodine as a super-stoichiometric oxidant. The catalyst system has precedent for also facilitating oxygenation reactions [82], which was observed as a competing pathway under these conditions.

The catalytic cycle proposed by the authors begins at resting state **I** (Figure 31), which is generated in situ and is subsequently oxidised to Mn(V)-oxo species **II** by hypervalent iodine oxidant PhIO. This can perform a HAT from the benzylic substrate, in turn generating a benzylic radical and Mn(IV)-hydroxy species **III**. Ligand exchange with the fluoride source affords complex **IV**, which performs FAT with the benzylic radical furnishing the desired product and regenerating **I**.

#### Photochemical methods

Photochemical methods that make use of fluoride to quench benzylic carbocations in order to form a new C–F bond have proved effective for functionalising a broad range of benzylic substrates. Two concurrent publications by the Doyle and Musacchio groups in 2021 and 2022 demonstrated the effective use of photochemical oxidative radical-polar crossover mechanisms to achieve this.

**Figure 32:**
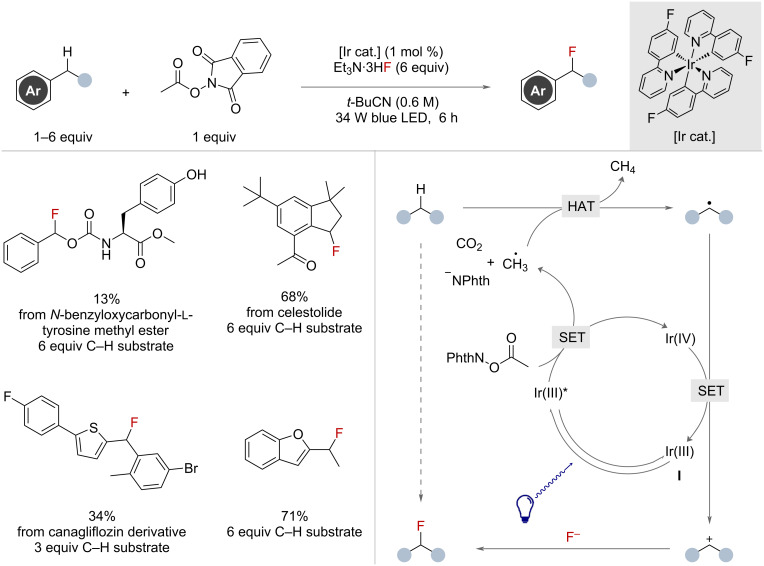
Iridium-catalysed photocatalytic benzylic C(sp^3^)–H fluorination with nucleophilic fluoride and *N*-acyloxyphthalamide HAT reagent.

The Doyle group reported the use of an iridium-catalysed system in this context with Et_3_N·3HF as the fluoride source (Figure 32) [83]. Photoexcitation of the Ir(III) catalyst **I** with blue light resulted in the photoexcited Ir(III)* catalyst, which was capable of performing a single-electron reduction on *N*-acyloxyphthalimide, promoting decarboxylation, releasing CO_2_, a methyl radical, anionic phthalimide and an Ir(IV) species. The resultant methyl radical displayed high affinity for benzylic HAT, in turn affording a benzylic radical and methane. The Ir(IV) species then oxidised the benzylic radical to the benzylic cation regenerating the ground-state iridium species, completing the catalytic cycle. Attack of the benzylic cation by fluoride, from Et_3_N·3HF, provided the benzylic fluoride product. Although a majority of examples were performed with an excess of benzylic substrate (up to 6 equivalents with respect to methyl radical precursor), a broad scope with excellent functional group tolerance was demonstrated. Difluorination was possible under these conditions, but required first generating the monobenzyl fluoride in situ from the corresponding benzyl chloride before undergoing the photochemical transformation to give the difluorination product. The authors showed that this HAT-radical-polar crossover approach could be applied to other nucleophiles, including water to give benzylic alcohols, or methanol to give methoxy products.

**Figure 33:**
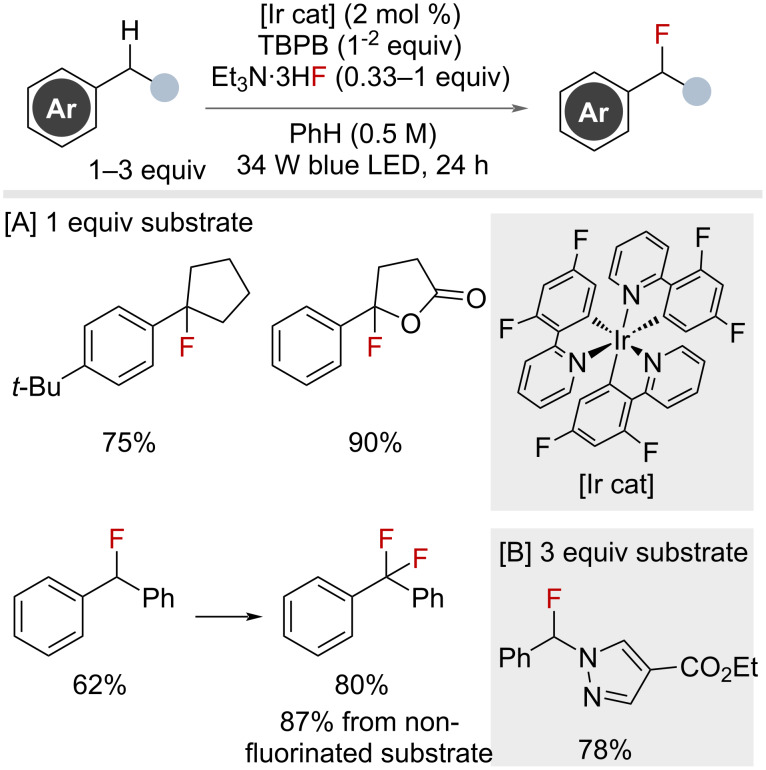
Iridium-catalysed photocatalytic benzylic C(sp^3^)–H fluorination with TBPB HAT reagent.

Musacchio and co-workers reported a similar approach for benzylic fluorination (Figure 33) [84], which followed a similar mechanistic blueprint to that reported by the Doyle group. Using *tert*-butoxide radicals, generated from reduction of *tert*-butyl benzoperoxoate (TBPB), selective benzylic HAT afforded the benzylic radical. Subsequent oxidation by Ir(IV) generated the benzylic cation that could be trapped by fluoride to afford the benzyl fluorides. An impressive scope with broad functional group tolerance, including bioactive molecules, was detailed in their work. Similar to the Doyle report, excess C–H substrate (up to 3 equivalents with respect to HAT reagent) was required in many cases, with the exception of tertiary benzylic substrates, which required only 1 equivalent of substrate and 2 equivalents of HAT reagent. Difluorination could be achieved using excess fluoride and HAT reagent. Other nucleophiles were amenable to the reaction conditions, allowing various benzylic functionalisation reactions, including acetoxylation and chlorination.

**Figure 34:**
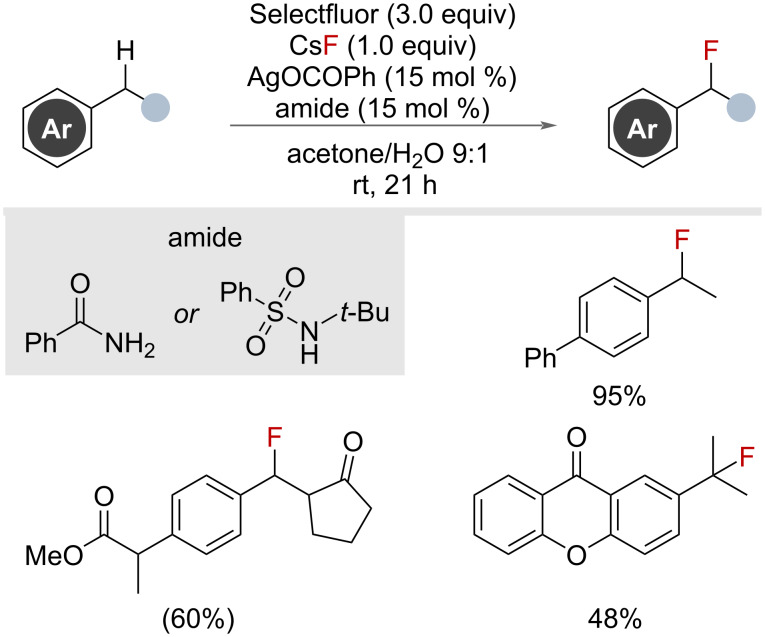
Silver-catalysed, amide-promoted benzylic fluorination via a radical-polar crossover pathway.

In 2023, Hamashima and co-workers disclosed an analogous, non-photochemical, silver-catalysed HAT radical-polar crossover mechanism for nucleophilic benzylic fluorination (Figure 34) [85]. The authors proposed a similar mechanistic pathway to the photochemical methods, citing the use of amide ligands as important for modulating the silver catalyst stability and oxidation potentials.

#### Electrochemical methods

**Figure 35:**
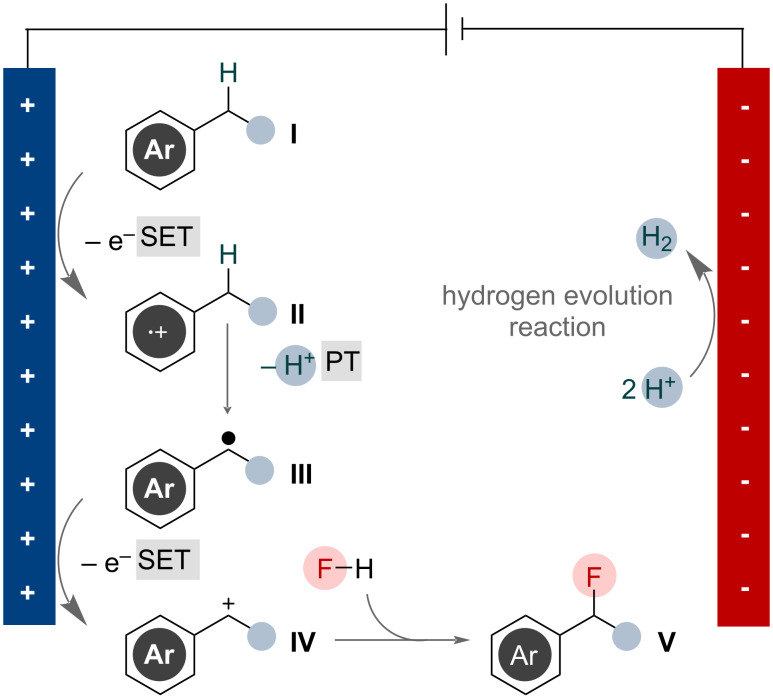
General mechanism for oxidative electrochemical benzylic C(sp^3^)–H fluorination.

Synthetic electrochemistry is a powerful tool offering excellent control over reaction kinetics and selectivity [86]. Electrochemical oxidation has been demonstrated as an efficient means for generating benzylic cations, allowing for the introduction of a host of functional groups [68]. This approach can also be applied for nucleophilic fluorination of benzylic substrates. This occurs via sequential electron-transfer and proton-transfer steps, as outlined in Figure 35 [87].

Single-electron oxidation of benzylic substrate **I** at the anode generates radical cation **II**. The acidity of benzylic protons is augmented after oxidation of the adjacent π-system, facilitating rapid proton transfer at this position, resulting in benzylic radical **III** [13,88]. Single-electron oxidation of the resulting benzylic radical is facile and expected to occur readily under the cell potentials required to initiate the first single-electron transfer, resulting in benzylic cation **IV** [89–90]. This species can then be captured by fluoride to give benzylic fluoride product **V**.

HF·amine ionic liquids are a popular choice of fluoride source in organic electrochemistry as their function is three-fold; as a fluoride source, as a supporting electrolyte and as a proton source, allowing for the hydrogen-evolution reaction as the counter electrode process [91]. Benzylic fluorination with these reagents has been observed as a side-product in the electrochemical generation of hypervalent fluoroiodane reagents [92–93].

**Figure 36:**
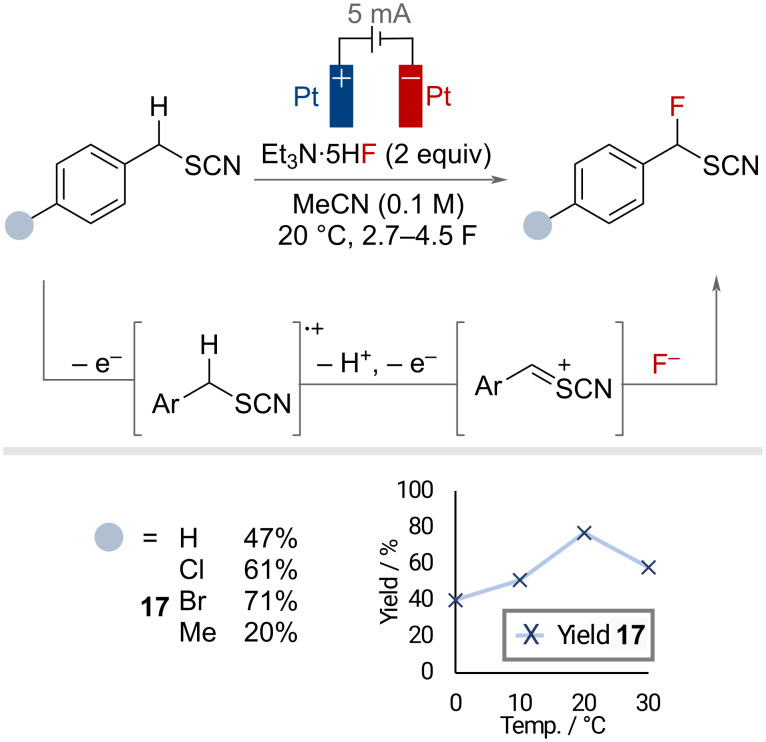
Electrochemical benzylic C(sp^3^)–H fluorination with HF·amine reagents.

In 2000, Fuchigami and co-workers demonstrated the effectiveness of these reagents in the oxidative electrochemical fluorination of benzylic positions adjacent to thiocyanate groups (Figure 36) [94]. The authors proposed anodic oxidation to generate a radical cation that can undergo facile α-proton elimination facilitated by the strongly electron-withdrawing thiocyanate group. Subsequent anodic oxidation affords a cationic species that can be trapped by fluoride to afford the product. This reaction was demonstrated on four substrates in yields of 47–71%. The authors noted a sensitivity to the fluoride source, with Et_3_N·5HF determined to be superior, and reaction temperature, as demonstrated by fluctuations in the yield of product **17** depending on the reaction temperature.

**Figure 37:**
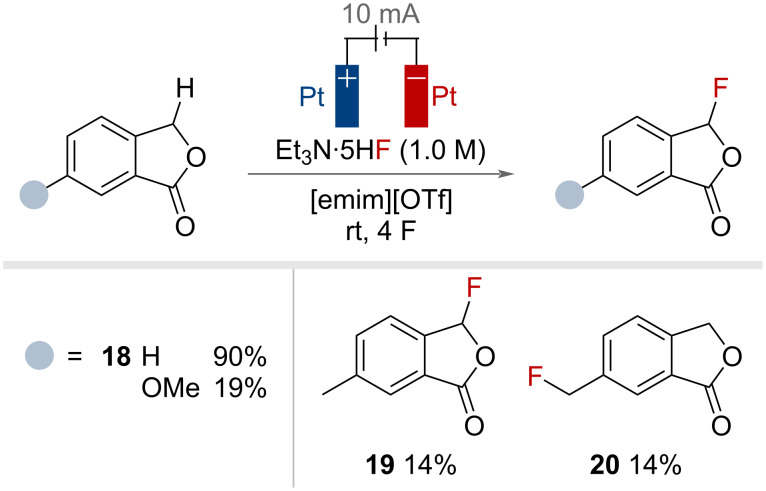
Electrochemical benzylic C(sp^3^)–H fluorination with 1-ethyl-3-methylimidazolium trifluoromethanesulfonate ([emim][OTf]) and HF·amine reagents.

In 2003, Fuchigami and co-workers also reported the use of Et_3_N·5HF in combination with the ionic liquid 1-ethyl-3-methylimidazolium trifluoromethanesulfonate ([emim][OTf]) for the fluorination of phthalides at the benzylic position (Figure 37) [95]. It was considered that the zwitterionic nature of the ionic liquid served two purposes. Firstly, to enhance the nucleophilicity of fluoride, and secondly, to improve the electrophilicity of the phthalide cationic intermediate generated by the SET/PT/SET sequence. Model substrate **18** could be fluorinated in excellent yield, but the yields decreased upon variation of the substrate. A poor selectivity for primary and secondary benzylic positions was observed when both positions were present, as highlighted by the formation of **19** and **20** in equal yields from the same substrate.

**Figure 38:**
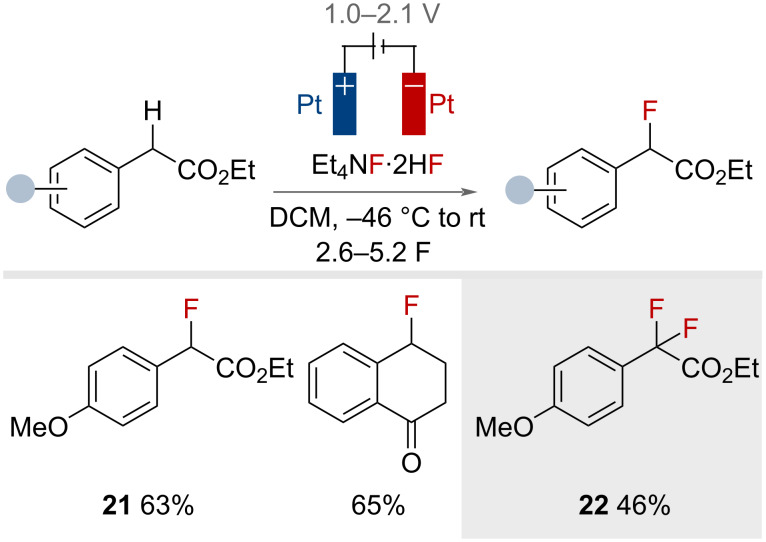
Electrochemical benzylic C(sp^3^)–H fluorination of phenylacetic acid esters with HF·amine reagents.

In the same year, Yoneda and co-workers reported the electrochemical benzylic fluorination of four phenylacetic acid esters and 1-tetralone (Figure 38) [96]. Et_4_N·2HF proved to be the best of the HF·amine reagents screened. The reaction was conducted under constant potential conditions, using cyclic voltammetry prior to electrolysis to determine the appropriate oxidation potential required for each substrate. Under these conditions, yields of up to 65% were achieved. Product **21** could be resubjected to the reaction conditions, affording difluoride **22** in 46% yield.

**Figure 39:**
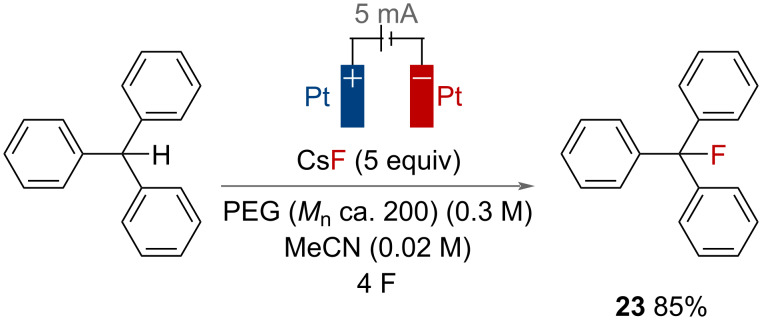
Electrochemical benzylic C(sp^3^)–H fluorination of triphenylmethane with PEG and CsF.

Metal fluorides are an economical source of nucleophilic fluorine, but are sparingly soluble in organic solvents. To overcome this, in 2012, Fuchigami and co-workers used polyethylene glycol (PEG) to dissolve caesium fluorides and facilitate an electrochemical benzylic C(sp^3^)–H fluorination of triphenylmethane (Figure 39) [97]. The authors suggested that PEG complexed the metal ion, increasing the nucleophilicity of the fluoride ion. Product **23** was achieved in 85% isolated yield after a small optimisation campaign.

**Figure 40:**
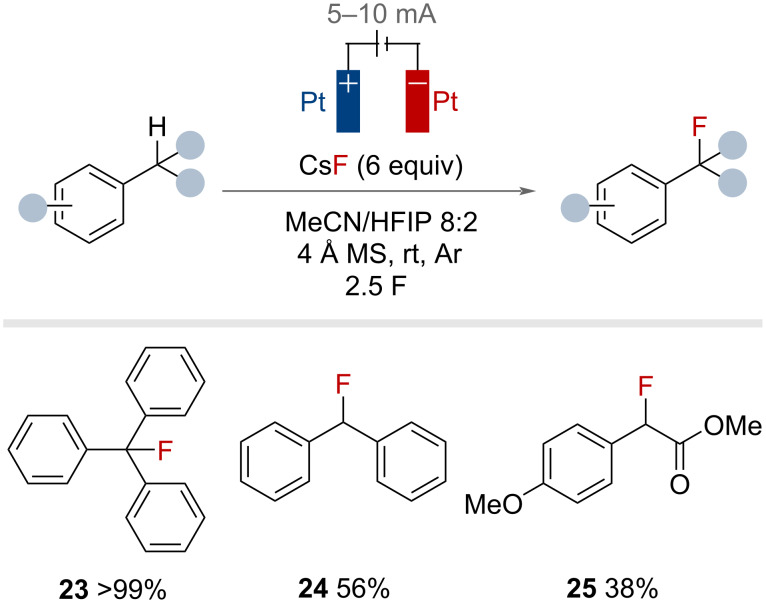
Electrochemical benzylic C(sp^3^)–H fluorination with caesium fluoride and fluorinated alcohol HFIP.

The fluorinated alcohol HFIP was used to dissolve caesium fluoride allowing for the electrochemical benzylic fluorination by Fuchigami, Inagi and co-workers in 2021 (Figure 40) [98]. The HFIP/CsF system functioned as both a fluoride source and as supporting electrolyte, enabling the passage of current through the reaction medium. Heavily stabilised **23** was afforded in quantitative yield. The protocol could be extended to other substrates to give **24** and **25**, albeit in reduced yields. The addition of molecular sieves and an atmosphere of argon ensured the best yields.

**Figure 41:**
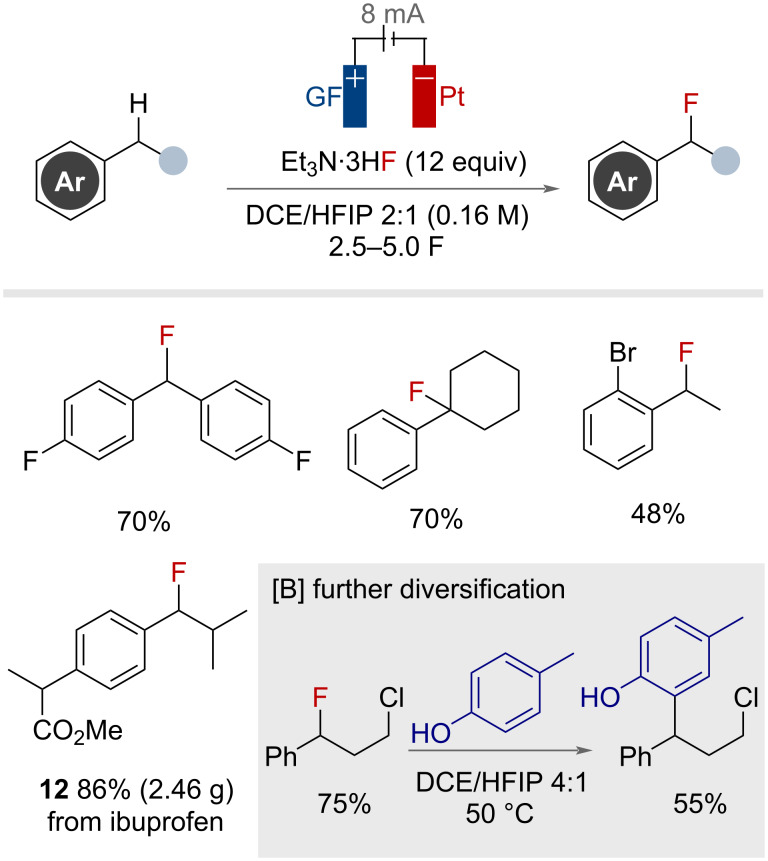
Electrochemical secondary and tertiary benzylic C(sp^3^)–H fluorination. GF = graphite felt. DCE = 1,2-dichloroethane.

Building on the work of Fuchigami, a more general electrochemical method for the nucleophilic fluorination of secondary and tertiary benzylic C(sp^3^)–H bonds was reported by Ackermann and co-workers in 2022 (Figure 41) [99]. A solvent mixture of DCE and HFIP (2:1) and 12 equivalents of Et_3_N·3HF resulted in the highest yields, with the authors proposing that HFIP aided in stabilising the electrochemically generated benzylic radical cation intermediates. Secondary and tertiary benzylic substrates bearing halogen, ester, protected amine and alkyl functional groups tolerated the reaction conditions well. The authors showed they were able to scale-up and selectively fluorinate the ibuprofen methyl ester at the methylene group to produce over 2 g of product **12**. The utility of the benzyl fluoride products as strategic intermediates for benzylation of electron-rich arenes was demonstrated by the authors (Figure 41B). Overall, this work demonstrates the broadest range of secondary and tertiary benzylic substrates for electrochemical nucleophilic fluorination.

**Figure 42:**
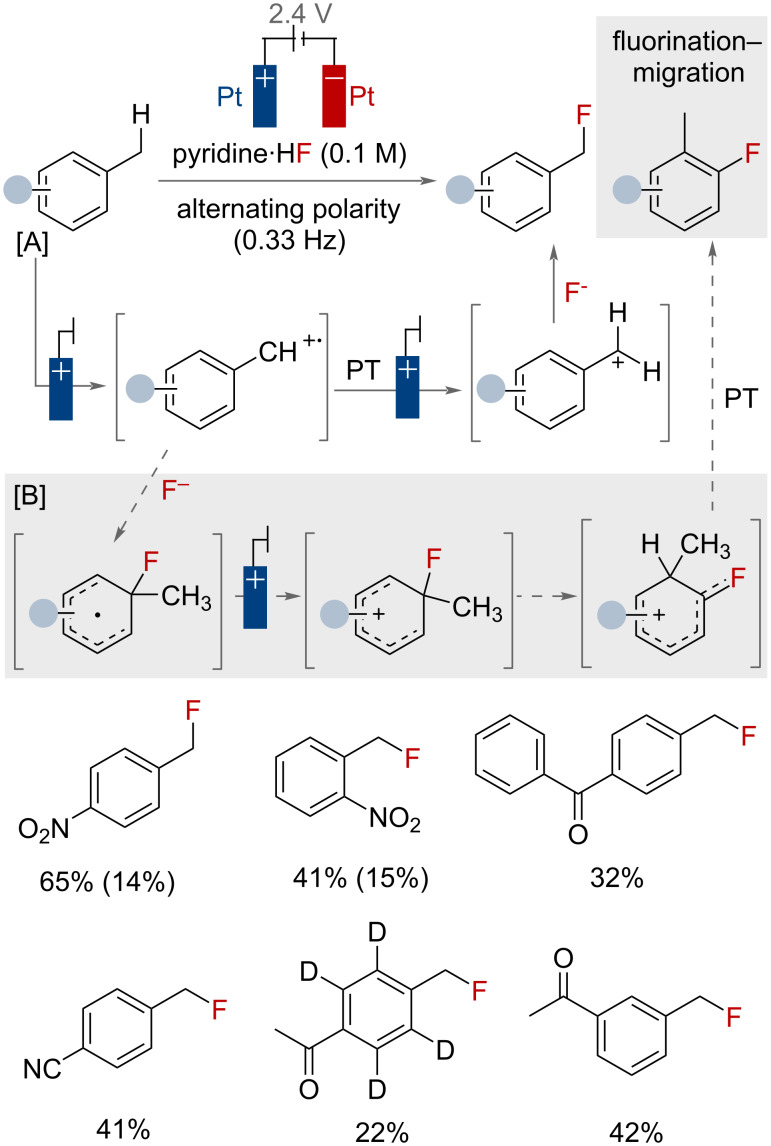
Electrochemical primary benzylic C(sp^3^)–H fluorination of electron-poor toluene derivatives. Ring fluorination–migration product yields in parentheses.

As highlighted by the previous examples, electrochemical oxidation is a useful tool for preparing benzylic fluorides. However, a number of reports highlight the fragility of secondary and tertiary benzyl fluorides, as they observe elimination and hydrolysis in many cases [20,100], thereby raising question marks over their suitability as synthetic targets. Monofluorinated methyl arenes, however, are much more stable to these decomposition pathways. The nucleophilic fluorination of primary benzylic substrates is a highly challenging reaction, due to the lower stability of the reactive intermediates involved in the mechanism. This is reflected in the fact that very few papers have been reported beyond the work on methylquinolines by the Sanford group (Figure 30), and a few preliminary electrochemical examples [93,101]. Middleton and co-workers described an alternating polarity approach for the fluorination of simple toluene derivatives in neat pyridine·HF (Figure 42) [102–103]. Poor conductivity necessitated the use of this waveform type. The benzylic fluorination was proposed to proceed via the classical ET/PT/ET pathway (pathway [A]). Nitro, cyano and sulphonyl fluoride substituents on the ring afforded ring fluorination–migration byproducts (via pathway [B]). In total, 14 substrates were fluorinated with yields ranging from 12–58%. Difluorination was observed under prolonged reaction times or upon increasing the applied cell potentials.

**Figure 43:**
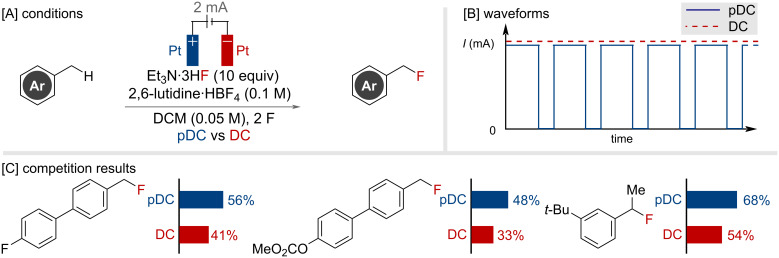
Electrochemical primary benzylic C(sp^3^)–H fluorination utilizing pulsed current electrolysis.

In 2024, Lennox and co-workers reported their investigation in exploring how alternative electrolysis waveforms might assist in the generation of reactive primary benzylic cations for nucleophilic fluorination (Figure 43) [104]. The challenge involved avoiding over-oxidation of the monofluorination product and overcoming mass transportation issues. It was found that the use of pulsed electrolysis waveforms, via the introduction of resting periods during electrolysis, was beneficial for the reaction outcome. This was demonstrated on a series of primary benzylic biphenyls and two secondary substrates by comparing to the pulsed technique (pDC) to the traditional direct current (DC) technique (Figure 43B and C). Under a constant potential (CP) regime no product was observed, but it was demonstrated that the introduction of a resting period, to generate a pulsed step–constant potential waveform (pSCP), assisted in the formation of benzyl fluoride product. The positive effect of the pulsed waveforms was attributed to a modulation of the electrical double layer, which results in improved mass transport, and subsequently decreases over-oxidation and decomposition to improve the reaction efficiency overall.

## Conclusion

The fluorination of benzylic C(sp^3^)–H bonds provides rapid access to an important functional group used in medicinal chemistry to control the pharmacokinetic profile of drug candidates. Historical and recent research efforts have resulted in a collection of protocols for the benzylic C(sp^3^)–H fluorination that demonstrate a broad tolerance of substrate classes. Electrophilic fluorination protocols are effective for specific substrate classes. Metal-catalysed processes operating via C–F reductive elimination pathways demonstrate stereospecificity, again on predefined substrate classes. Radical fluorination methods offer an expansion to substrate scopes and rely on the use of more expensive fluorine-atom-transfer reagents. Finally, oxidative benzylic activation methods, often in tandem with enabling technologies, such as photoredox catalysis and electrochemistry, open up the use of nucleophilic fluoride sources, complementing the broader scopes demonstrated by radical methods. All these approaches highlight the multiple reactivity modes of benzylic C(sp^3^)–H bond functionalisation, and provide context on the state of the art and will hopefully encourage further development in key areas. This is particularly pertinent to the late-stage benzylic fluorination of complex molecules, which will require exceptionally mild conditions in order to tolerate a broad range of functional groups.

## Data Availability

Data sharing is not applicable as no new data was generated or analyzed in this study.
